# Single-cell transcriptome atlas reveals spatiotemporal developmental trajectories in the basal roots of moso bamboo (*Phyllostachys edulis*)

**DOI:** 10.1093/hr/uhad122

**Published:** 2023-06-09

**Authors:** Zhanchao Cheng, Changhong Mu, Xiangyu Li, Wenlong Cheng, Miaomiao Cai, Chongyang Wu, Jutang Jiang, Hui Fang, Yucong Bai, Huifang Zheng, Ruiman Geng, Junlei Xu, Yali Xie, Yuping Dou, Juan Li, Shaohua Mu, Jian Gao

**Affiliations:** Key Laboratory of National Forestry and Grassland Administration/Beijing for Bamboo & Rattan Science and Technology, International Center for Bamboo and Rattan, Beijing 100102, China; Key Laboratory of National Forestry and Grassland Administration/Beijing for Bamboo & Rattan Science and Technology, International Center for Bamboo and Rattan, Beijing 100102, China; Key Laboratory of National Forestry and Grassland Administration/Beijing for Bamboo & Rattan Science and Technology, International Center for Bamboo and Rattan, Beijing 100102, China; Key Laboratory of National Forestry and Grassland Administration/Beijing for Bamboo & Rattan Science and Technology, International Center for Bamboo and Rattan, Beijing 100102, China; Key Laboratory of National Forestry and Grassland Administration/Beijing for Bamboo & Rattan Science and Technology, International Center for Bamboo and Rattan, Beijing 100102, China; Key Laboratory of National Forestry and Grassland Administration/Beijing for Bamboo & Rattan Science and Technology, International Center for Bamboo and Rattan, Beijing 100102, China; Key Laboratory of National Forestry and Grassland Administration/Beijing for Bamboo & Rattan Science and Technology, International Center for Bamboo and Rattan, Beijing 100102, China; Key Laboratory of National Forestry and Grassland Administration/Beijing for Bamboo & Rattan Science and Technology, International Center for Bamboo and Rattan, Beijing 100102, China; Key Laboratory of National Forestry and Grassland Administration/Beijing for Bamboo & Rattan Science and Technology, International Center for Bamboo and Rattan, Beijing 100102, China; Key Laboratory of National Forestry and Grassland Administration/Beijing for Bamboo & Rattan Science and Technology, International Center for Bamboo and Rattan, Beijing 100102, China; Key Laboratory of National Forestry and Grassland Administration/Beijing for Bamboo & Rattan Science and Technology, International Center for Bamboo and Rattan, Beijing 100102, China; Key Laboratory of National Forestry and Grassland Administration/Beijing for Bamboo & Rattan Science and Technology, International Center for Bamboo and Rattan, Beijing 100102, China; Key Laboratory of National Forestry and Grassland Administration/Beijing for Bamboo & Rattan Science and Technology, International Center for Bamboo and Rattan, Beijing 100102, China; Key Laboratory of National Forestry and Grassland Administration/Beijing for Bamboo & Rattan Science and Technology, International Center for Bamboo and Rattan, Beijing 100102, China; Key Laboratory of National Forestry and Grassland Administration/Beijing for Bamboo & Rattan Science and Technology, International Center for Bamboo and Rattan, Beijing 100102, China; Key Laboratory of National Forestry and Grassland Administration/Beijing for Bamboo & Rattan Science and Technology, International Center for Bamboo and Rattan, Beijing 100102, China; Key Laboratory of National Forestry and Grassland Administration/Beijing for Bamboo & Rattan Science and Technology, International Center for Bamboo and Rattan, Beijing 100102, China

## Abstract

Roots are essential for plant growth and development. Bamboo is a large Poaceae perennial with 1642 species worldwide. However, little is known about the transcriptional atlas that underpins root cell-type differentiation. Here, we set up a modified protocol for protoplast preparation and report single-cell transcriptomes of 14 279 filtered single cells derived from the basal root tips of moso bamboo. We identified four cell types and defined new cell-type-specific marker genes for the basal root. We reconstructed the developmental trajectories of the root cap, epidermis, and ground tissues and elucidated critical factors regulating cell fate determination. According to *in situ* hybridization and pseudotime trajectory analysis, the root cap and epidermis originated from a common initial cell lineage, revealing the particularity of bamboo basal root development. We further identified key regulatory factors for the differentiation of these cells and indicated divergent root developmental pathways between moso bamboo and rice. Additionally, *PheWOX13a* and *PheWOX13b* ectopically expressed in *Arabidopsis* inhibited primary root and lateral root growth and regulated the growth and development of the root cap, which was different from *WOX13* orthologs in *Arabidopsis*. Taken together, our results offer an important resource for investigating the mechanism of root cell differentiation and root system architecture in perennial woody species of Bambusoideae.

## Introduction

The global bamboo forest area, also known as ‘the world’s second largest forest’ has reached 32 million hm^2^, and is developed and utilized by ~2.5 billion people in various ways [1–3]. There are 1642 species of bamboo worldwide, including ~100 species used for commercial purposes [2]. In 2020, the total international trade in bamboo products was valued at USD 2.21 billion [4]. The output value of the bamboo industry in China reached 321.7 billion RMB in 2020 [5]. According to the agenda of 12 consecutive United Nations Climate Change Conferences from COP15 to COP27, BARC (Global Bamboo and Rattan Congress) 2018 and 2022 [6–8], bamboo has made great contributions to climate change mitigation, carbon peaking, and green sustainable development. Moso bamboo (*Phyllostachys edulis*), accounting for ~70% of the total bamboo growth area, has developed as a representative plant in fundamental research on Bambusoideae [9]. Moso bamboo has a comprehensive carbon sequestration capacity, which is 1.46 times that of China fir (*Cunninghamia lanceolata*) forest and 1.33 times that of tropical rainforest under the same conditions [10–12]. The underground absorption, transportation, and storage system of moso bamboo forest is composed of parts including the bamboo rhizome, rhizome roots, and basal roots [13]. Rhizome roots and basal roots are crucial for water absorption and energy flow during the rapid growth period of bamboo, which can grow >20 m tall within 45–60 days [14]. For a long time, studies focused on the aboveground part of bamboo, while underground systems, especially root systems, have received little theoretical investigation [15–18].

**Figure 1:**
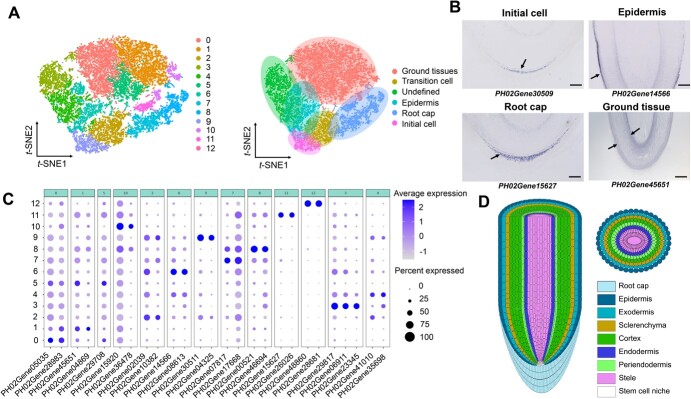
Single-cell clusters in moso bamboo basal roots. (A) Two *t*-SNE plots of ~14 279 filtered cells of moso bamboo basal root showing 13 clusters in four different tissues with additional subclusters. Cluster 9 represents the initial cell; Cluster 2 represents the transition cell; Clusters 7, 8, 11, and 12 represent the root cap; Cluster 6 represents the epidermis; Clusters 0, 1, 5, and 10 represent ground tissues; Clusters 3 and 4 represent undefined tissues. (B) Representative marker genes for the *in situ* hybridization of four putative clusters in moso bamboo basal roots. Scale bars, 300 μm. (C) Expression patterns of representative cell-type marker genes across cell clusters. Dot diameter shows the proportion of cluster cells expressing a given gene; color shows expression across cells in the cluster. Details of all marker genes are given in Supplementary Data Table S2. (D) Schematic of anatomy of moso bamboo basal roots.

The root systems of most dicotyledonous plants, such as *Arabidopsis*, consist of a single primary root and numerous lateral roots. In contrast, monocotyledons, including wheat, maize, and rice, generate a fibrous root system containing numerous postembryonic adventitious roots from the stem base. In addition, basal roots, as a type of adventitious root generated at the base of bamboo, grow with the rapid growth of the bamboo and gradually become lignified and wither when the bamboo stops growing. Bamboo roots mainly consist of the root cap, epidermis, cortex, and vascular column, which is similar to *Arabidopsis* and rice [16, 19, 20]. However, bamboo is usually grown under well-watered and well-drained conditions and has generated a distinct anatomy to adjust to this environment. The internal structures of bamboo root organization differ greatly from those of rice and *Arabidopsis*. For example, the ground tissue of bamboo roots is composed of dozens of layers of cortical cells, which constitute the outer cortex, peripheral fibrous tissue, cortical parenchyma, air cavity, periendodermis, and endodermis (Fig. 1D, Supplementary Data Fig. S1). The periendodermis is the outer layer of cells around the inner cortex; the cells are spherical, flat spherical, or polygonal. Its cell walls are thickened, except the tangential plane, which is similar to the endodermis, forming a C-shaped thickening, but the thickening degree is obviously weaker than that of the endodermis. The increased cortical layer number and special periendodermis structure of bamboo play key roles in adaptation to moist conditions [16, 18]. Overall, the anatomy of bamboo roots has been investigated, but the molecular definition and distribution of different types of cells are largely largely unknown.

At present, single-cell RNA sequencing (scRNA-seq) for several plant species, such as *Arabidopsis* [19, 21], rice [22, 23], tomato [24], maize [25], peanuts [26], *Populus* [27–29], tea [30], and woodland strawberry [31], has been reported, confirming a high rate of heterogeneity of plant tissues as well as special marker genes for different cell types, cell division, and cell differentiation [32]. However, for non-model plants, including bamboo, it is difficult to identify cell specificity and analyze the function of key genes due to the lack of marker genes for cell type and an efficient and stable genetic transformation system [33–35]. Moreover, the difficulty of protoplast isolation and the complexity of cell types have resulted in little progress in scRNA-seq application in plants. A method for extraction of protoplasts from 15-day-old bamboo leaf sheaths was developed [36]. The protoplasts of root could also be obtained by this method, but there were abundant starch granules and dead cells in the protoplast suspension, which made it difficult to obtain high-quality protoplasts. Fortunately, we constructed a purification technique for bamboo root protoplasts, which was able to remove a large number of starch granules and dead cells, making it possible to apply scRNA-seq to bamboo roots.

In this study we present a high-resolution single-cell transcriptome atlas of moso bamboo basal roots that captures precise spatiotemporal information. Combining scRNA-seq analyses with *in situ* hybridization, four cell types were identified by novel cell-type-specific marker gene key regulators and revealed the heterogeneity of moso bamboo basal root cells. Our transcriptome atlas offers an unparalleled spatiotemporal developmental trajectory of basal root cell-type differentiation at a single-cell resolution. Comparative analyses of root cap and epidermis initial cell lineage differentiation between bamboo and rice revealed divergent features of basal root development and further improve the understanding of root development and the evolutionary relationship between annual and perennial monocots.

## Results

### scRNA-seq and identification of moso bamboo basal root cell clusters

Protoplast isolation of moso bamboo root used a highly efficient protocol for leaf sheath protoplast preparation of bamboo [35]. In order to reduce the effect of protoplasting treatment, we shortened the enzyme digestion time from 4 to 3 hours. The proportion of viable cells increased from ~72.6 to 86.3% (Supplementary Data Fig. S2). About 3.7 × 10^8^ living protoplasts were isolated per gram. The basal root cells of moso bamboo with rapid growth are packed with many starch granules, which is a great challenge for scRNA-seq. After separating the moso bamboo root protoplasts, 20% sucrose solution was carefully placed into the bottom of the centrifuge tube containing the protoplast suspension. After centrifugation for 1 minute at 50 g/min, most of the dead cells and debris fell into the sucrose solution and gathered at the bottom of the centrifuge tube, while viable cells floated at the upper and lower interfaces. Healthy protoplasts floating at the upper and lower interfaces were transferred into a clean centrifuge tube (Supplementary Data Fig. S2). The percentage of live cells reached >85%, which complied with the criteria for scRNA-seq.

We obtained ~15 324 cells from two biological replicates of basal root tips (Supplementary Data Table S1). The single-cell DNA sequencing libraries were made and sequenced using Illumina NovaSeq 6000. Two biological replicates were carried out. The sequencing processed a total of 14 279 filtered cells from two samples, and obtained a median 1753 and 1964 genes per cell respectively (Supplementary Data Table S1, Supplementary Data Fig. S3). Using the FindClusters function (resolution = 0.8), these unsupervised analyses grouped root cells into 13 cell clusters (Fig. 1A).

Because almost no marker genes are currently available for bamboo root cell types, the following two strategies were used to identify different cell clusters in the moso bamboo cell atlas. First, we performed *in situ* hybridization histochemistry using orthologs of rice and *Arabidopsis* root marker genes to annotate some clusters (Supplementary Data Table S2) [19, 21–23, 37–40]. However, the transcriptional levels of most orthologs of marker genes were different from those in rice and *Arabidopsis* and were not well detected using *in situ* hybridization and scRNA-seq data as a result of their low expression (Supplementary Data Figs S4, S5, and S6). We also found that most of these genes for different cell types of *Arabidopsis* and rice roots did not show specific expression for cell type in moso bamboo basal roots (Supplementary Data Figs S4, S5, and S6). A pool of known marker genes might be divergent among *Arabidopsis*, rice, and bamboo and hence were not suitable for cross-species cell-type identification. Second, we examined gene expression patterns on UMAP (uniform manifold approximation and projection) and *t*-SNE (*t*-distributed stochastic neighbor embedding) to identify these cell clusters. In order to identified novel marker genes, the top 10 differentially expressed genes (DEGs) in 13 cell clusters were used to produce a heat map describing their diverse expression profiles (Supplementary Data Fig. S7). These novel marker genes were used for making a distinction between each cell type in moso bamboo basal root tip. In addition, we selected some potential marker genes with high and specific expression in one or two clusters and used *in situ* hybridization assays to confirm special cell-type cluster annotations (Fig. 1B and C, Supplementary Data Fig. S4). The results enabled us to assign four major cell types to moso bamboo basal roots. For example, *PH02Gene04325* and *PH02Gene30509* were specifically expressed in the meristematic cell clusters (initial cells) of moso bamboo basal roots (Cluster 9), *PH02Gene17668*, *PH02Gene46694*, *PH02Gene15627*, and *PH02Gene28681* in the root cap cells (Clusters 7, 8, 11, and 12, respectively), *PH02Gene10382* in the columella root cap cells and epidermis cells (Cluster 2), *PH02Gene14566* in the epidermis (Cluster 6), and *PH02Gene45651*, *PH02Gene04869*, *PH02Gene29708*, and *PH02Gene15920* in the ground tissues (Clusters 0, 1, 5, and 10, respectively) (Fig. 1B, Supplementary Data Fig. S4). Of note, differentiation trajectories, such as those of the root cap and epidermis, radiated away from the meristematic cell clusters by UMAP. Overall, we identified four distinct cell clusters that corresponded to four major cell types and a number of novel bamboo-specific marker genes.

### Pseudotime trajectory of root cap cell development

**Figure 2:**
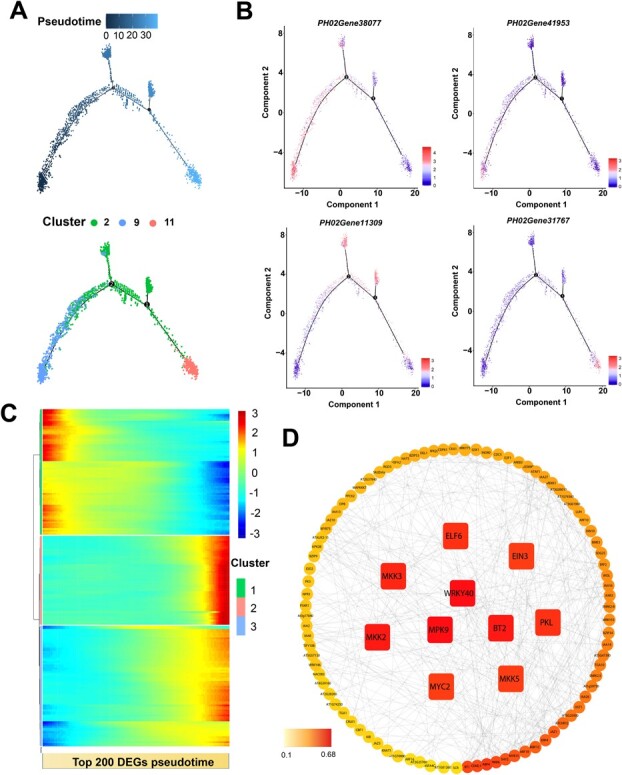
Differentiation trajectories of basal root cap cells. (A) A continuous differentiation trajectory from initial cell to basal root cap cell was obtained using Monocle 2. Each dot shows an individual cell. The colors represent the pseudotime score. Blue, green, and red represent Clusters 9, 2, and 11, respectively. (B) Expression of root cap cell-type-specific genes (*PH02Gene38077*, *PH02Gene41953*, *PH02Gene11309*, and *PH02Gene31767*) along with the pseudotime trajectory. The colors on the dots represent the transcriptional levels of these genes in individual cells. (C) A heat map displaying the expression pattern of branch-dependent genes in root cap cells along pseudotime. Each row indicates one gene. The bar represents the relative expression level. (D) The interaction network of representative genes was constructed by using their homologous genes in *Arabidopsis*.

The root cap of the plant is located at the front of the apical meristem zone, and not only protects the meristem zone, but may also be a necessary response to environmental signals, such as gravity, humidity, and light. Because cells undergoing intermediate states can be captured, scRNA-seq makes it possible to explore continuous differentiation trajectories of diverse developmental processes. Therefore, we first inferred the developmental trajectory of moso bamboo root cap cells. Re-clustering of Clusters 2, 11, and 9 showed six sub-cell clusters, named 0–5 (Supplementary Data Fig. S8). Interestingly, consistent with the distribution distance on the UMAP, plotting revealed that initial cells (Cluster 9) were first developed into Cluster 2, which further developed into root cap tissue cells (Cluster 11) (Supplementary Data Fig. S8). According to Monocle 2, the differentiation time analysis revealed that the cells in Clusters 2 and 11 were more differentiated than those in Cluster 9 (Fig. 2A). Therefore, these results demonstrated that those cells in Cluster 9 served as root cap initial cells. These cells gradually differentiated into columella root cap cells and lateral root cap cells with cell division and differentiation. A total of 200 top DEGs were identified along the pseudotime trajectory and were divided into three clusters with different gene expression profiles reflecting transcriptional signature during root cap development (Fig. 2C). For instance, *PH02Gene38077*, *PH02Gene41953*, *PH02Gene11309*, and *PH02Gene31767* were selected as representative genes for the pseudotime trajectory of Clusters 9, 2, and 11, respectively (Fig. 2B). The scRNA-seq dataset analysis revealed that the transcription of *PH02Gene38077* and *PH02Gene41953* gradually decreased over the pseudotime trajectory, followed by increased expression of *PH02Gene11309* and *PH02Gene31767* (Fig. 2B). Moreover, 1123 transcription factors (TFs) were identified in the differential expression profile (Supplementary Data Table S3). To further discover hub genes involved in cell fate determination, an interactive network was performed using orthologous TFs of *Arabidopsis*. The analyses indicated that MKK2/3/5, WRKY40, MPK9, MYC2, PKL, ELF6, and EIN3 consisted of a core interaction network (Fig. 2D). PKL/SSL2 regulated lateral root formation in *Arabidopsis* by inhibiting auxin [41, 42]. The chromatin-remodeling gene *AtCHR12* mediates in plant growth and development upon perceiving environmental stress [43]. To better explain this molecular regulatory mechanism underlying root cap differentiation, we performed Gene Ontology (GO) analyses (Supplementary Data Table S4) and heat map analyses of the representative differentially expressed TFs (Supplementary Data Fig. S9). Cell differentiation is the process in which cells undergo a series of physiological, biochemical, and morphological changes related to phenylpropanoid metabolism and its metabolites. Interestingly, we observed that Clusters 2, 9, and 11 were all enriched in the phenylpropanoid biosynthetic process and lignin catabolic process according to GO analysis. Cluster 9 was significantly enriched in cell differentiation, plant-type cell wall organization, regulation of root morphogenesis, and auxin biosynthetic process. Cluster 2 was mainly enriched in the regulation of root morphogenesis, and negative regulation of the cytokinin-activated signaling pathway, while Cluster 11 was specifically enriched in response to auxin, regulation of cell size, and the gibberellin biosynthetic process. In addition, gene expression patterns varied widely between clusters. Collectively, genes related to the root cap terminal and intermediate developmental states were found, indicating that those genes may regulate cell state transition.

### Differentiation trajectory of epidermal cells

The root epidermis generally consists of a layer of epidermal cells with a protective function. Based on functional annotations, Cluster 6 was designated as the epidermis, and Clusters 2, 7, 8, 11, and 12 belonged to the root cap (Fig. 1). Interestingly enough, the distribution distances on the UMAP and *t-*SNE plots revealed that the root cap and epidermis cell clusters were connected to Cluster 9 (Fig. 1A, Supplementary Data Fig. S10), which were annotated as initial cells (Fig. 1B). Therefore, the epidermis and root cap cells of moso bamboo may be from the same undifferentiated progenitor cells. However, in dicots the root cap and epidermis cells belong to a common initial cell, while in monocots the ground tissues and epidermis cells originate from a single common initial cell [44].

**Figure 3:**
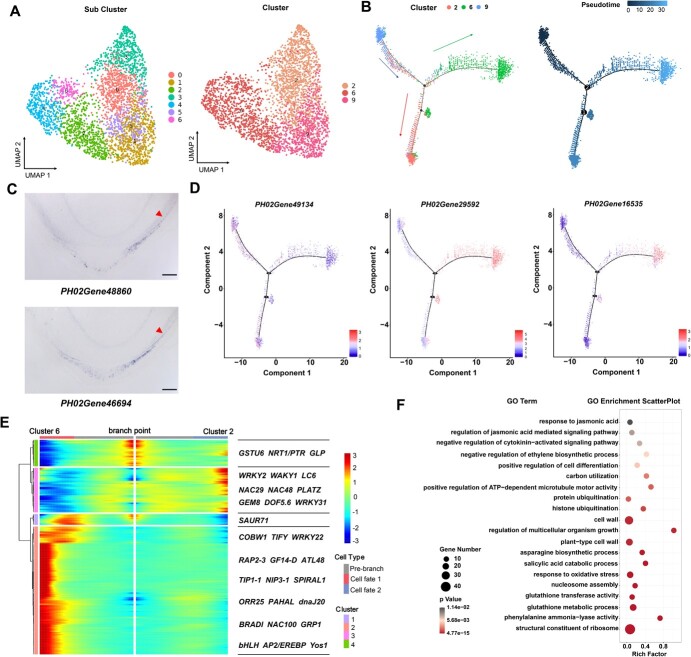
Differentiation trajectories of basal root epidermis cells. (A) UMAP showing root epidermis cell populations (Clusters 2, 6, and 9). 0–6, sub-cell clusters. The line indicates a potential differentiation trajectory from the initial cells to the root epidermis cells. (B) A continuous differentiation trajectory from initial cell to epidermis cell was obtained using Monocle 2. Each dot shows an individual cell. The colors represent the pseudotime score. Blue, red, and green represent Clusters 9, 2, and 6, respectively. (C) *In situ* hybridization validation of *PH02gene48860* and *PH02Gene46694* in basal root tip. Scale bars, 300 μm. (D) Expression of epidermis cell-type specific genes (*PH02Gene49134*, *PH02Gene29592*, and *PH02Gene16535*) along with the pseudotime trajectory. (E) Heat map displaying the expression pattern of branch-dependent genes in epidermis cells along pseudotime. The right of the heat map shows representative genes. Cluster 2 genes are enriched in cells on the prebranch and branch 1 of the trajectory, and Cluster 1 and 3 genes are enriched in cells on branch 2 of the trajectory. Each row represents one gene. The bar represents the relative expression level. (F) Scatter plots of GO enrichment analysis for Clusters 9, 2, and 6. Only enriched categories (*P* value <.05) are shown.

To test this hypothesis, we reconstructed the developmental trajectory relationship between the root cap and the epidermis. Clusters 2, 6, and 9 were selected to produce a bifurcate pseudotime backbone with three distinct final states (Fig. 3B), where the clusters were arranged along different branches. The developmental trajectory started from the initial cells (Cluster 9) and ended in two branches: root cap cells (Cluster 2) and epidermal cells (Cluster 6). In addition, *in situ* hybridization analyses preliminarily confirmed that the representative genes *PH02Gene04325* and *PH02Gene30509* in Cluster 9 were specifically expressed in the linkage region comprising columella root cap stem cells and epidermal initial cells (Fig. 1B, Supplementary Data Fig. S4). Root cap representative genes, such as *PH02Gene15627*, *PH02Gene48860*, *PH02Gene17668*, and *PH02Gene46694*, had slight expression in very few cells of the epidermis (Figs 1B and 3C, Supplementary Data Fig. S4).

Moreover, we identified the top 500 significant DEGs along the pseudotime trajectory and generated an expression heat map of these genes, which together showed four distinct clusters. Gene expression profiles varied greatly among three branching points. Particularly, some of these genes were known to play significant roles in root development (Fig. 3E, Supplementary Data Table S3). For instance, germin-like protein GLP (*PH02Gene49134*) was prominently expressed at the branching point and neighboring cells (Fig. 3D, Supplementary Data Table S5), in agreement with its important role in the development of plant embryos and in the modification and reconstruction of cell walls [45]. *PH02Gene29592*, associated with root development, lateral root development, and root hair cell tip growth, was preferentially expressed in the epidermis branch (Fig. 3D, Supplementary Data Table S5). Interestingly, NAC29 and NAC48 were related to multicellular organism development, which were highly expressed in root cap (Fig. 3E). In addition, *PH02Gene08613*, highly expressed in the epidermis branch, was initially enriched in functional categories, such as the defense response to fungus and cuticle development (Supplementary Data Table S5). These representative genes were enriched in GO terms related to response to plant-type cell wall and positive regulation of cell differentiation, which improves understanding of the hypothesis mentioned above (Fig. 3F, Supplementary Data Table S6). Taken together, the above results provide insights into the differentiation trajectory of the root cap and epidermis cells. Further molecular and genetic experiments are needed to verify their function in the differentiation of the root cap and epidermis.

### Differentiation trajectories of ground tissue cells

**Figure 4:**
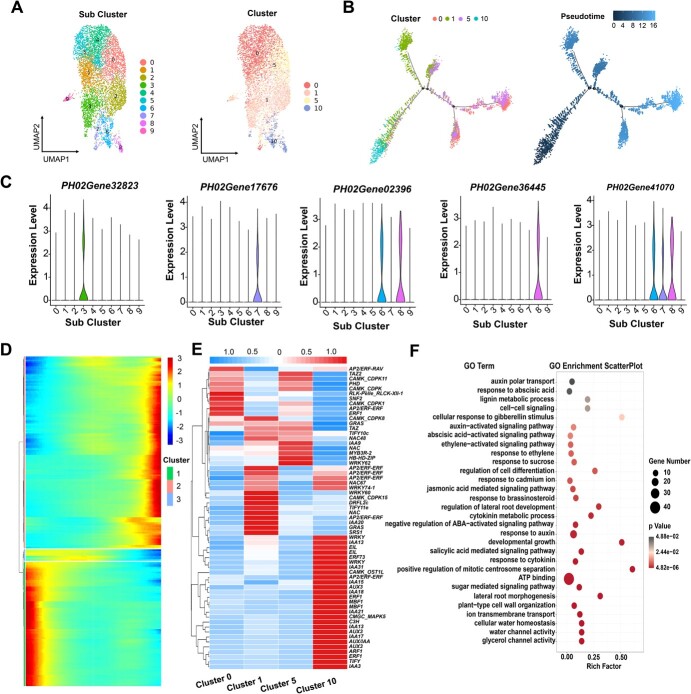
Differentiation trajectories of basal root ground tissue cells. (A) UMAP projections showing root ground tissue cell populations (Clusters 0, 1, 5, and 10). 0–9, sub-cell clusters. The line indicates a potential differentiation trajectory from the initial cells to the root ground tissue cells. (B) A continuous differentiation trajectory over ground tissue cells was obtained using Monocle 2. Each dot shows an individual cell. The colors represent the pseudotime score. Red, olive green, purple, and blue represent Clusters 0, 1, 5, and 10, respectively. (C) Violin graph showing expression patterns of *PH02Gene32823*, *PH02Gene17676*, *PH02Gene02396*, *PH02Gene36445*, and *PH02Gene41070* (orthologous genes in rice) in 10 subclusters (A). The violin represents the proportion of cells expressed in the subcluster. (D) Heat map displaying the expression pattern of branch-dependent genes in ground tissue cells along pseudotime. Each row represents one gene. The bar shows the relative expression level. (E) Heat map displaying the expression pattern of representative TFs related to plant hormone pathways. (F) Scatter plots of GO enrichment analysis for clusters 0, 1, 5, and 10. Only enriched categories (*P* value <.05) are shown.

The plant root ground system serves as a barrier structure to regulate the flow diffusion of ions, which is crucial for the absorption of water and mineral nutrients. Successive divisions of the bamboo ground tissue initial cell generate the exodermis, sclerenchyma layers, parenchymatous cells, periendodermis, and endodermis [16, 18]. Reclustering of the four clusters belonging to the ground system population (Clusters 0, 1, 5, and 10) revealed 10 sub-cell clusters, named 0–9 (Fig. 4A). In addition, some orthologs of rice known marker genes including *PH02Gene32823*, *PH02Gene17676*, *PH02Gene02396*, *PH02Gene36445*, and *PH02Gene41070* involved in the ground tissue were identified among diverse sub-cell clusters (Fig. 4C) [23].

To better understand the gene regulatory basis of ground tissue differentiation, the cells from Clusters 0, 1, 5, and 10 were reclustered and pseudotime trajectory and heat map analyses were performed. We delineated four distinct cell lineages, leading to a four-forked pseudotime backbone representing four distinct final states (exodermis, sclerenchyma cell layer, periendodermis, and endodermis) (Fig. 4B). Interestingly, pseudotime analysis revealed that inferred developmental trajectories confirmed gradual transitions from early cells in Cluster 10 to late ground tissue cells (Clusters 0, 1, and 5) (Fig. 4B). Therefore, these analyses collectively demonstrated that those cells in Cluster 10 may serve as initial cells of ground tissue. To further identify genes patterns, we displaying the top 200 DEGs expressed along the pseudotime trajectory of ground tissue cells with three distinct clusters (Fig. 4D, Supplementary Data Table S3). Genes were upregulated or downregulated in different clusters, indicating their important role in regulating cell differentiation. Some key genes underlying their differentiation showed temporal expression patterns along the pseudotime trajectory, such as *PIP1;1* (ortholog of *PH02Gene00514*), expressed at the early stage, acting as an active water channel protein, and playing vital physiological roles in roots [46].

We selected all specific TFs based on their high expression levels in a specific cell type and high fold changes compared with other clusters. A total of 231 putative TFs were identified in ground tissue of moso bamboo, of which 61 were identified as being involved in the plant hormone pathways (Supplementary Data Table S7, Fig. 4E). For instance, AUX/IAA and ARF, related to the auxin pathway, were preferentially expressed in Cluster 10. However, other TFs, such as AP2/EREF WYKY, DOF, and MYB, associated with ethylene, jasmonic acid, and abscisic acid-activated signaling pathways, respectively, were highly enriched in Clusters 0, 1, and 5. Further GO analyses of representative DEGs indicated that a number of genes were highly enriched in water channel activity, regulation of cell differentiation, lateral root morphogenesis, and response to cadmium ion, supporting ground tissue involvement in the flow diffusion of ions, water, and mineral nutrients (Fig. 4F, Supplementary Data Table S6). These results provide a better understanding of the developmental mechanisms of basal root ground tissue, which will be useful in the future.

### Overexpression of *PheWOX13a* and *PheWOX13b* inhibited root growth in *Arabidopsis*

In the above sections, we report many TFs in different types of basal root cells in moso bamboo. To establish their functionality in basal root growth, the expression levels of all TFs were checked in our single-cell transcriptomes of basal roots, as well as published transcriptome profiles [47–51]. WOXs play an important role in maintenance of the root and stem apical meristem. Our bulk transcriptome data indicated that *PheWOX13a* and *PheWOX13b*, which were remarkably orthologous to *OsWOX13* and *AtWOX13*, had higher expression than other WOX genes in root tissues [47, 52, 53]. In addition, based on single-cell expression profiles, *PheWOX13a* and *PheWOX13b* had higher expression in Cluster 10 and Cluster 12 than other clusters (Supplementary Data Fig. S11).

**Figure 5:**
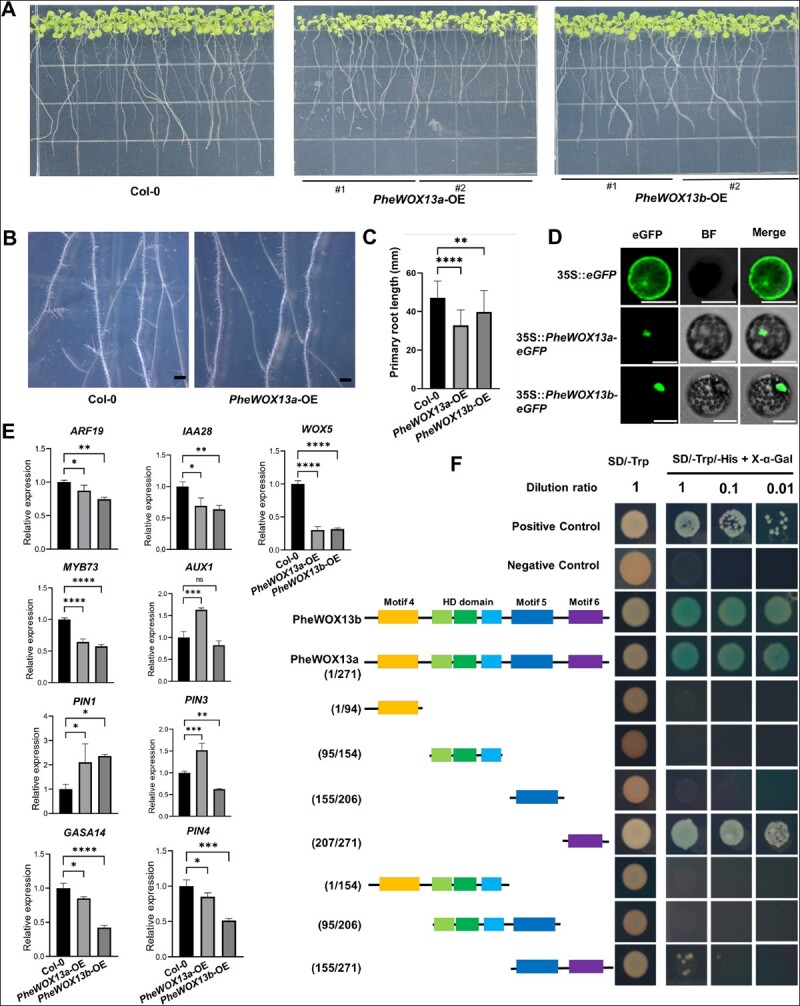
Effects of *PheWOX13a* and *PheWOX13b* overexpression on *Arabidopsis* root development. (A) Comparative primary root growth of *PheWOX13a* and *PheWOX13b* transgenic lines and Col-0 (wild-type) seedlings in the first 12 days of development. (B) Representative primary roots and lateral roots from the Col-0 and *PheWOX13a*-OE lines. Scale bar = 1000 μm. (C) Quantification of primary root length in (A). Data are mean ± standard error (*n* = 30 roots). (D) Subcellular localization of PheWOX13a-eGFP and PheWOX13b-eGFP in protoplasts of *Arabidopsis* leaves. eGFP, eGFP fluorescence; BF, bright field; Merge, merge of eGFP and BF images. Scale bars = 50 μm. (E) Relative expression levels of genes related to root and lateral root development in Col-0, *PheWOX13a*-OE, and *PheWOX13b*-OE. *Actin 2* served as the reference gene. Data are mean ± standard error (*n* = 3). Asterisks indicate a statistically significant difference between Col-0 and transgenic plants (*t*-test, ^*^P < .05, ^**^P < .01, ^***^P < .001, ^****^P < .0001, ns, no significant difference). (F) Transcriptional activation analysis of *PheWOX13a* and *PheWOX13b* through a yeast one-hybrid system. Transcriptional activation activity of different truncations of *PheWOX13a* were performed. Numbers on the left side show different positions of amino acids in PheWOX13a. The right side shows growth of the transformants with three different dilution rates of 1, 0.1, and 0.01 on SD/−Trp and SD/−Trp/−His adding 50 mM 3-AT. The ꞵ-galactosidase activities were examined by X-Gal staining. Positive control: PGBKT7–53 + PGADT7-T; negative control: pGBKT7.

Subsequently, we focused on the molecular functions of *PheWOX13a* and *PheWOX13b*. We overexpressed *PheWOX13a* and *PheWOX13b* in *Arabidopsis* because the efficiency of the genetic transformation of moso bamboo is much lower than that in *Arabidopsis* [35, 54]. At the 10-day seedling stage, overexpression of *PheWOX13a* and *PheWOX13b* resulted in shorter primary root growth compared with Col-0 (Fig. 5A and B), suggesting that *PheWOX13a* and *PheWOX13b* negatively regulate primary root development. Compared with Col-0, *PheWOX13a*-overexpressing (OE) lines exhibited a significant reduction in emerged lateral roots (LRs) through optical microscopic observation (Fig. 5C). Moreover, lateral root densities of *PheWOX13a*-OE and *PheWOX13b*-OE lines were significantly lower than that of wild-type (Supplementary Data Fig. S12). *PheWOX13a* overexpression in *Arabidopsis* inhibited root cap growth, while *PheWOX13b* promoted abnormal root cap development (Supplementary Data Fig. S13). To investigate the role of *PheWOX13a* and *PheWOX13b*, we examined the transcript levels of genes associated with root development. Compared with Col-0, the expression of auxin synthesis-related genes *YUC1*, *YUC2*, *YUC6*, and *TAA1* in *PheWOX13a*- and *PheWOX13b*-OE lines did not change significantly (Supplementary Data Fig. S14). Interestingly, the expression of *PIN3* and *AUX1* related to auxin transport was significantly upregulated in the *PheWOX13a* transgenic lines, while it was downregulated in the *PheWOX13b* transgenic lines. Overexpression of *PheWOX13a* and *PheWOX13b* resulted in a significant increase in *PIN1* expression and a clear reduction in *PIN4* expression. In addition, *PheWOX13a* and *PheWOX13b* might directly or indirectly downregulate the mRNA level of *WOX5*, which plays a crucial role in determining the fate of stem cells [55]. *GASA14* was speculated to function in the promotion of cell elongation by gibberellic acid, which was significantly downregulated in *Arabidopsis* ectopically expressing *PheWOX13a* and *PheWOX13b*. In addition, several lateral root regulatory genes, including *AUXIN RESPONSE FACTOR ARF19*, early auxin response gene *IAA28*, and *MYB73* [56–58] in transgenic lines, showed significantly lower transcription levels than those in Col-0 (Fig. 5E).

To further investigate the subcellular localization of *PheWOX13a* and *PheWOX13b*, they were fused to GFP under control of the CaMV 35S promoter and then transferred into *Arabidopsis* and moso bamboo protoplast cells, respectively. Transiently expressed PheWOX13a and PheWOX13b proteins were specifically localized in the nuclei (Fig. 5D, Supplementary Data Fig. S15). The transactivation abilities of PheWOX13a and PheWOX13b were analyzed by a yeast assay system. X-gal staining for β-galactosidase activity showed that PheWOX13a and PheWOX13b had transcriptional activation ability (Fig. 5F). In addition to the conserved HD DNA-binding domain, the WOX13 protein in Poaceae contained three specific domains, Motif 4, Motif 5, and Motif 6 [59]. To determine the transcriptional activation domain of PheWOX13a, we made seven constructs containing each domain [amino acid numbers 1–94 (1/94), 95/154, 155/206, 207/271, 1/154, 95/206, 155/271] in pGBKT7-BD vector. This result showed that the transcriptional activation domain of PheWOX13a was located at the C-terminal region (207/271) containing Motif 6. A dual-luciferase (LUC) reporter assay was used to determine the transcriptional activation ability of PheWOX13a in *Nicotiana benthamiana*. PheWOX13a promoted more relative activity of firefly LUC than that in the control, indicating that PheWOX13a has transcriptional activity (Supplementary Data Fig. S16). Combination analysis of subcellular localization and transcriptional activation indicated that PheWOX13a can function as a transcription activator. In conclusion, we hypothesized that *PheWOX13a* and *PheWOX13b* have different molecular regulatory mechanisms controlling root growth via the auxin pathway, which differs from orthologous *WOX13* in *Arabidopsis*. These results provide new insights into the molecular functions of *PheWOX13a* and *PheWOX13b*.

## Discussion

Rhizome roots and basal roots play an important role in fixing, absorbing, and transporting water and nutrients during the rapid growth period of bamboo. Unlike the roots of *Arabidopsis* and rice, which have been extensively investigated, rhizome roots and basal roots of bamboo have been studied only at the anatomical level. scRNA-seq has been utilized to reveal cell heterogeneity, discover new marker genes, and describe the developmental trajectory. In our study, a modified protocol for protoplast preparation was constructed to remove many secondary metabolites and promote the percentage of live cells, which made the high-throughput application of scRNA-seq in bamboo roots possible. By identifying tissue-specific markers and different cell types (Fig. 1), the first single-cell transcriptome atlas for moso bamboo root development was generated in perennial Poaceae plants. The differentiation trajectories of the root cap, epidermis, and ground tissue with cellular expression profiling were described in detail. In addition, DEG analysis and cell annotation for differentiation trajectories reveal key regulatory genes that may reflect the cell fate direction. Overall, scRNA-seq has been applied to bamboo as a non-model plant, and this finding is beneficial for facilitating future research on Bambusoideae plant development at the single-cell resolution.

Compared with *Arabidopsis* and rice, there is a lack of a complete understanding of bamboo root development. Although the anatomical structure of bamboo roots has been preliminarily studied, the molecular definition of its cell types is incomplete. Moreover, it is difficult to infer bamboo root cell types based on homologous *Arabidopsis* and rice gene expression due to differences in the number and type of cell types between dicots and monocots. Based on bamboo root origins, it can be divided into two categories: rhizome roots emerging from the rhizome node and basal roots generated from the base node of the shoot. The basal roots bear a strong resemblance to crown roots, differentiating from the nodes of the main stem and tillers. However, bamboo and rice each prefer strikingly different survival conditions, which might cause a large difference among cell types, cell quantity, and cellular structure. For example, bamboo root ground tissue contains a periendodermis, which is absent in *Arabidopsis* and rice. Moreover, bamboo root cortex can differentiate into special aerenchyma with an anatomical adaptation to waterlogging, which is found in rice but absent from *Arabidopsis*. To obtain a better understanding of how root stem cells generate diverse cell types, we successfully defined four cell types in the bamboo basal root tip based on a combination of scRNA-seq analysis and *in situ* hybridization. Cell clusters corresponding to the root cap, epidermis and ground tissue were also identified. Unfortunately, cell types belonging to the periendodermis and other undefined cell types were not identified in our scRNA-seq dataset, perhaps due to the low number of marker genes or the sequencing depth and gene coverage in our scRNA-seq datasets. Importantly, novel marker genes for these root cell types were identified by *in situ* hybridization assays (Fig. 1, Supplementary Data Fig. S4). In future research, it may be possible to define more cell types and discover more special marker genes through spatial transcriptomics and laser capture microdissection single-cell PCR.

In the root meristematic zone, diverse cell types are derived from one or more stem cells located at the root apical area. Along with stem cell division, tissue-specific transition cells (TACs) are gradually generated. The apical meristem of most dicots has three columns of protocells: the mesocolumn protocell in the upper layer, the cortical protocell in the middle layer, and the common protocell of the epidermis and root cap in the lower layer, which are occasionally present in a small number of monocots. For most monocots, the root cap has an independent protocell, while the epidermis and cortex originate from the cortical protocell together. For instance, the ground tissue and epidermis share a single common initial cell in the monocot rice, whereas the lateral root cap and epidermis belong to a common initial cell in the dicot *Arabidopsis* [22]. The mechanism by which these different cell fates are progressively determined from an ordinary stem cell is largely unknown. Our results showed that Cluster 6 was designated as epidermis, and Clusters 2, 7, 8, 11 and 12 belonged to the root cap. The root cap and epidermis cell clusters were connected to Cluster 9 as the initial cell type by the UMAP and *t*-SNE plots (Fig. 1A, Supplementary Data Fig. S10), indicating that Cluster 9 might be composed of mixed meristematic cells generating a distinct root cap and epidermis cell types. In addition, based on *in situ* hybridization analyses, the special expression patterns of representative genes in the root cap and epidermis cells further demonstrated that the root cap and epidermis belonged to a common initial cell lineage. The identification of initial cell marker genes for the root cap and epidermis in bamboo transgenic systems and cell fate localization will help to accurately map the differentiation trajectory of cell lineages in specific tissues in the future.

In vascular plants, WOXs play an important role in the maintenance of the root and stem apical meristem, vascular development, embryogenesis and development, and adventitial organogenesis and development [60–63]. The *WOX13* gene has been reported to promote replum formation in *Arabidopsis* [64]. *WOX13* plays a vital role in cellular reprogramming during stem cell initiation in the moss *Physcomitrella patens* [65]. Moreover, WOX13 acts as a key regulator for callus formation and organ reconnection in *Arabidopsis* [66]. In this study, we identified *PheWOX13a* and *PheWOX13b* (orthologous genes of *WOX13*) as key regulators that orchestrate the expression of genes involved in primary root and lateral root growth (Fig. 5). This was in accordance with our phenotypic analysis of the *PheWOX13a-* and *PheWOX13b-*OE lines. Based on dot plots representing scRNA-seq analysis, *PheWOX13a* was preferentially enriched in Cluster 10, belonging to the ground tissues of basal roots, while *PheWOX13b* had high expression in Cluster 12 as part of the root cap. In *Arabidopsis*, overexpression of *PheWOX13a* and *PheWOX13b* showed shorter primary roots. Specifically, the expression levels of auxin synthesis-related genes (*YUC1*, *YUC2*, *YUC6*, and *TAA1*) showed no change between wild-type and transgenic lines, indicating that *PheWOX13a* and *PheWOX13b* were unable to regulate root growth by affecting auxin synthesis genes. In comparison with the wild type, there were several auxin transport genes, such as *PIN3* and *AUX1*, showing the opposite expression patterns between the *PheWOX13a-* and *PheWOX13b-*OE lines. In addition, overexpression of *PheWOX13a* and *PheWOX13b* resulted in the expression reduction of WOX5, which was a critical regulator in maintaining stem cell fate, revealing that *PheWOX13a* and *PheWOX13b* might control root development by regulating *WOX5* expression. However, we have shown that *PheWOX13a*-OE lines developed short roots or a rootless phenotype, likely because of the low expression of lateral root regulatory genes, such as *ARF19*, *IAA28*, and *MYB73* [56–58], which were dependent on the auxin pathway. We hypothesized that the overexpression of *PheWOX13a* and *PheWOX13b* in transgenic *Arabidopsis* may regulate auxin accumulation and distribution in primary and lateral roots by regulating the expression of genes involved in auxin transport and response, thereby simultaneously inhibiting primary root elongation and lateral root formation. In addition, a new specific domain, Motif 6, located at the C-terminal of WOX13, existed widely in monocots (such as rice, maize, and five bamboo species), showing species specificity [59]. Our transcriptional activation analysis further indicated that Motif 6 enabled PheWOX13a to function as a transcriptional activator. Overall, the functions of *PheWOX13a* and *PheWOX13b* in bamboo differed from those in the previously reported orthologous gene *WOX13* in *Arabidopsis*. However, further studies are required to confirm the regulatory mechanism of *PheWOX13a* and *PheWOX13b* involved in root growth.

In our study, we robustly identified most of the major cell types of moso bamboo and defined new cell-type-specific marker genes. By time analysis of individual basal root tip cells, we reconstructed the sequential developmental trajectory of the root crown, epidermis, and ground tissue and elucidated the key candidate factors that determine cell fate in these cell lineages. Based on *in situ* hybridization and pseudotime locus analysis, the root crown and epidermis were derived from a common initial cell lineage, revealing the specificity of bamboo root development. *PheWOX13a* and *PheWOX13b* regulate primary root and lateral root growth by regulating the auxin pathway. Therefore, our results provide a valuable resource for studying the development and physiological function of cell types at the molecular level and at single-cell resolution in bamboo perennial woody plants.

## Materials and methods

### Plant materials and growth conditions

Basal roots of moso bamboo with 50-cm tall shoots were collected from Guangde City (30°49′13″ N, 119°25′28″ E), Anhui Province, China. Basal root tips (~2.0 cm long from the root tip) were gathered for scRNA-seq, as well as *in situ* hybridization.

Wild-type *Arabidopsis* (Col-0) and transgenic lines (transgenic homozygous lines) were used for qPCR and phenotypic assays. All seeds were sterilized with 75% alcohol and germinated on vertical square plates with 1/2 Murashige and Skoog (MS) at 23°C (day)/19°C (night) with long days (16 hours light/8 hours dark, with a light intensity of 85 mmol/m^2^/s). The seedlings of *Arabidopsis* were photographed and then analyzed after 12 days of growth.

### Basal root protoplast extraction and preparation for scRNA-seq

The basal root tips (~2 cm) were washed clean and cut into 1–2 mm strips, which were added to a sterile Petri dish with 25 ml sterile enzyme solution (3% cellulase R-10, 0.7% macerozyme R-10, 0.2% pectinase Y-23, 0.3% hemicellulose, 0.1% BSA, 0.01 M CaCl_2_, 20 mM MES, and 0.6 M mannitol) in a vacuum pump at 23°C for 20–30 minutes, and then the mixture was placed in an incubator with shaking at 65 rpm for 3 hours at 23°C to release basal root protoplasts. These protoplasts were filtered with a 40-μm cell strainer and then centrifuged at a speed of 100 *g* for 2 minutes at 23°C, and after the supernatant was sucked out 0.6 M mannitol was added to resuspend all protoplasts. After separating root protoplasts, they needed to be purified by sucrose density gradient centrifugation. The total viability of protoplasts needed to reach >85%, which was further confirmed with trypan blue staining. The concentration of basal root protoplasts was adjusted to 1000 cells/μl.

### scRNA-seq library construction and sequencing

After the protoplast suspensions were put into the 10 X Chromium instrument, the library was constructed using the 10 X Genomics Chromium Single Cell 3′ kit (V3). The specific experimental operation steps were implemented according to the scRNA-seq experimental manual of LC-Bio Technology (Hangzhou, China). The Illumina NovaSeq 6000 was used to sequence the library, which demanded a minimum sequencing depth of 20 000 reads per cell.

### Preprocessing of raw scRNA-seq data

The Cell Ranger (version 5.0.1) was used for sample demultiplexing, barcode processing, and single-cell 3′ gene counting, and scRNA-seq data were aligned to the Moso Genome (http://172.26.100.143/#/) [67] using STAR. Combined with the annotation information of the Moso Genome (GTF file), the reads aligned to the genome were divided into exons, introns, and intergenic regions (at least 50% of the bases were aligned to exons, introns, and intergenic regions of reads).

### Cell clustering and identification of marker genes

Cell Ranger analysis showed that 15 324 cells were captured from two basal root samples, some of which had low activity or were even dead cells. Cell filtration was performed using the data analysis R package of Seurat software (version 3.1.1) [68]. In all, 14 279 cells passed the quality control threshold: the number of genes identified was >500, the number of UMI (Unique Molecular Identifiers) was <500, and the expression ratio of mitochondrial DNA-derived genes was <25% in single cells; genes expressed in at least one cell should be retained.

To visualize the scRNA-seq data, the steps included the expression value of genes, calculated by the LogNormalize method of the Normalization function of the Seurat software. The normalized expression value was used to perform PCA (Principal Component Analysis). Within all principal components (PCs), clustering and *t*-SNE analyses were performed using the top 20 PCs [69]. According to the weighted shared nearest neighbor (SNN) module, cell clusters were annotated by an optimized clustering algorithm. The Find All Markers toolkit of Seurat was applied to analyze the DEGs between different cell clusters by the Wilcoxon rank-sum test with the following parameters: genes expressed in >10% of the cells in a cluster, and an average log (fold change) >0.25.

### Pseudotime analysis

Monocle 2 was used to perform pseudotime analysis on the scRNA-seq data of basal roots. First, Monocle identifies DEGs according to pseudotime values using the differentialGeneTest function. Next, we reduced the space down to one with two dimensions (max_components = 2, method = ‘DDRTree’), which were easily visualized and interpreted, while Monocle ordered the cells. Then, we ordered the cells using the orderCells function. Once the cells were ordered, we visualized the trajectory using plot_cell_trajectory in the reduced dimensional space. In addition, we need to call orderCells again with the root_state parameter to specify the beginning. Monocle used the Branched Expression Analysis Modeling (BEAM) method to analyze the cell data after pseudotime and the designated nodes to mine the DEGs related to branches, and used the plot_generes_branched_heapmap function to visualize the genes that obviously depend on branches.

### Protein–protein interaction network analysis

The protein–protein interaction network (PPI) network of the differentially expressed TFs (*P* < .05) in the pseudotime analysis was structured using their orthologs in the *Arabidopsis* genome on the STRING database (https://cn.string-db.org/), and Cytoscape (3.9.1) was used for visualization. Furthermore, we selected the top 100 TFs of degree as the core genes.

### Functional analysis

After selecting key genes during cell development, the R package was used for the GO enrichment analysis (http://geneontology.org). For convenience of display, only corrected *P*-values (statistical significance level of enrichment analysis) <.05 for enrichment items in the GO function enrichment analysis results were displayed. In addition, ggplot2 was used to visualize the GO enrichment analysis results.

### *In situ* hybridization

The specific DNA probes of the selected marker genes were designed and synthesized using GENWIZ (Supplementary Data Table S8). The basal root tips were harvested and immediately placed in 4% paraformaldehyde solution for fixation. The hybridization and immunological methods were performed as described previously [47, 48]. The images were collected using a Zeiss microscope (Axio Image.M2).

### Toluidine blue staining

The paraffin section method for basal root tissues was described previously [52]. The staining results were imaged on a Zeiss microscope (Axio Image.M2).

### qRT–PCR analysis

The total RNAs of Col-0 and transgenic lines were extracted by TRIzol reagent (Invitrogen, USA). Reverse transcription was performed with First-Strand Synthesis Master Mix (LABLEAD, China). According to the candidate gene sequence, specific primers were designed with Primer 5.0 (Supplementary Data Table S8). The qRT–PCR methods were described previously [48]. *Actin 2* was used as the reference gene. The relative expression levels for the target genes were calculated by the 2^-ΔΔCt^ method [70].

### Functional verification of transgenic plants

The coding sequences of *PheWOX13a* and *PheWOX13b* were inserted into *Actin*::*PheWOX13a* and *Actin*::*PheWOX13b* vectors. The vectors were introduced into *Agrobacterium tumefaciens* GV3101 and used for *Arabidopsis* transformation. The Stable overexpression lines of *PheWOX13a* and *PheWOX13b* were obtained in the *T*_3_ generation using kanamycin. The *T*_3_ generation seeds were cultured vertically on MS medium for 12 days, and the root lengths were counted with IMAGE J software. In addition, the root microstructures were observed using a Zeiss body microscope (Stemi-305). Using the DNA of transgenic plants as a template, the expression levels of representative genes related to root development were determined using qPCR with three biological repeats.

### Subcellular localization and transactivation assays

The full-length cDNAs of *PheWOX13a* and *PheWOX13b* were inserted into GFP protein driven by the CaMV 35S promoter. For transient expression assays, *Arabidopsis* and moso bamboo protoplasts were isolated and transfected according to the Sheen laboratory’s protocol for protoplast isolation [36, 71]. GFP protein fluorescence signals were obtained by a confocal laser scanning microscope (Zeiss Microsystems). Primer sequences used to generate fusion genes are listed in Supplementary Data Table S5.

The PCR products of *PheWOX13a* and *PheWOX13b* were cloned into the pGBKT7 DNA-BD vector containing the GAL4 DNA-binding domain. The recombinant vectors were transformed into the yeast strain AH109 containing the *His-3* and *LacZ* reporter genes, which was used to detect transcriptional activation. Primer sequences for cloning the full region of *PheWOX13a* and *PheWOX13b* are listed in Supplementary Data Table S5.

The full-length sequence of *PheWOX13a* was fused with the GAL4 DNA-binding domain, which was driven by the CaMV 35S promoter in the pEAQ-BD vector (as an effector). The reporter contained a firefly *LUC* fused with five GAL4-binding elements and a TATA box, and the *Renilla LUC* gene under the control of 35S promoter was regarded as the reference. Transient transformation in *N. benthamiana* and luminescence measurements were conducted by referring to previous studies [72, 73]. The primers used are listed in Supplementary Data Table S8.

## Supplementary Material

Web_Material_uhad122

## Data Availability

All high-throughput sequencing data have been deposited in the GEO under accession number GSE229126.
