# Supplementary material for: Single-cell transcriptome atlas reveals spatiotemporal developmental trajectories in the basal roots of moso bamboo (Phyllostachys edulis)
Source: Hortic Res. 2023 Jun 9;10(8):uhad122. doi: 10.1093/hr/uhad122 (PMC10405134; doi:10.1093/hr/uhad122)
Supplement: Web_Material_uhad122 [file web_material_uhad122.zip › Revised All supplemental Figures.pdf]

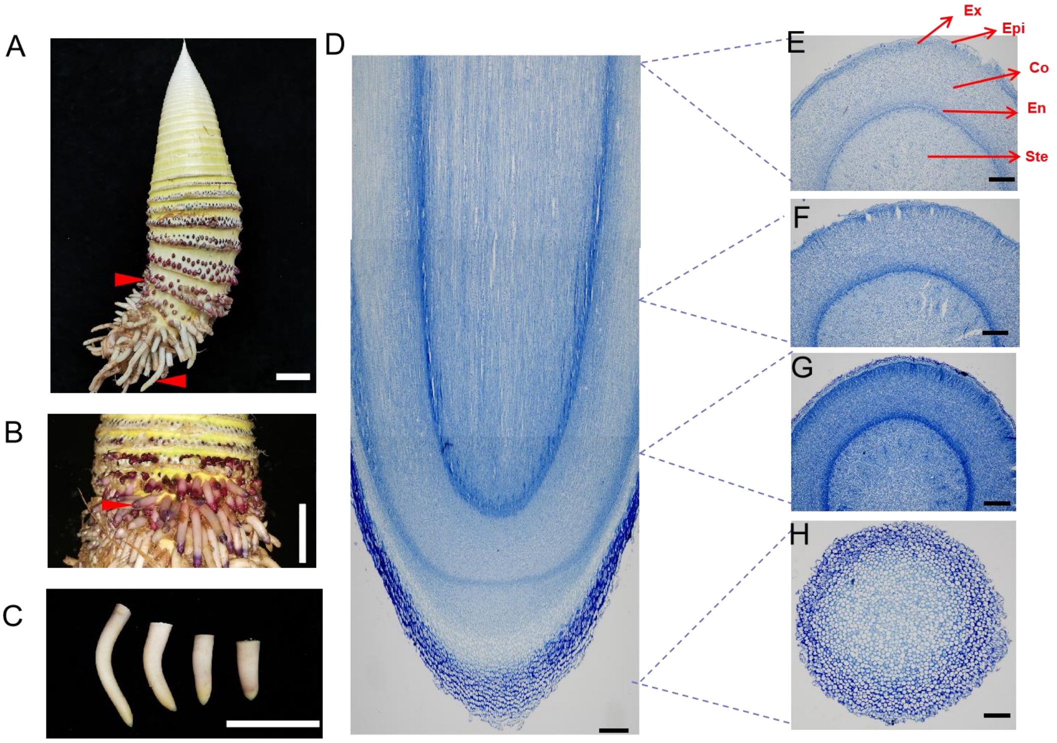

Supplemental Fig. S1 Root tips in Moso bamboo basal root.

(A-C) Basal roots of 50cm bamboo shoot. ► indicates basal root samples for scRNA-seq and in situ hybridization. Scale bars, 50mm. (D) Longitudinal section of basal root stained with toluidine blue.Scale bars, 300 μm. (E-H) Cross sections of basal root stained with toluidine blue. E-H correspond to mature zone, elongation zone, meristem zone and root cap in d. Scale bars, 300 μm. Epi: epidermis; Ex: exodermis; Co: cortex; En: Endodermis; Ste: stele.

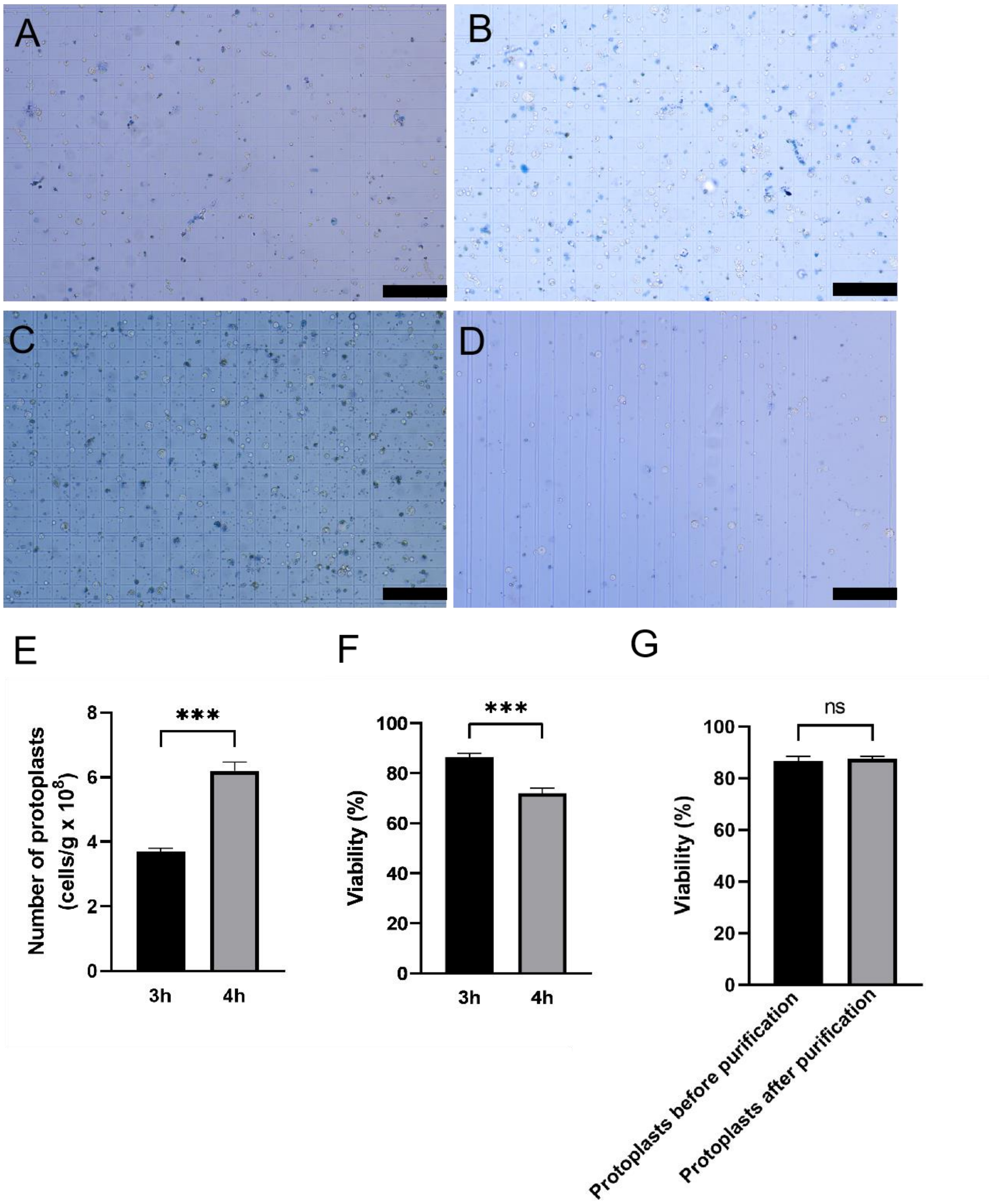

Supplemental Fig. S2 Isolation and purification of protoplasts from Moso bamboo root.

(A) Protoplasts incubated in enzyme solution for 3 h. (B) Protoplasts incubated in enzyme solution for 4 h. (C) Protoplasts before purification in 0.6 mannitol solution. (D) Protoplasts after purification in 0.6 mannitol solution. (E) Comparison of protoplast yield from different plasmolysis time. (F) Comparison of protoplasts viability from different plasmolysis time. (G) Comparison of protoplasts viability

between sucrose gradient centrifugation (Protoplasts after purification) and control group (Protoplasts before purification). Protoplasts purification was performed with sucrose gradient centrifugation at concentration of 20%, with the centrifugal speed of 50 g/min, and the centrifugal time of 1 min. Protoplasts viability was also evaluated by trypan blue exclusion. Protoplasts images were obtained using an ordinary optical microscopy with a 10 x magnification. Scale bars, 200 μm.

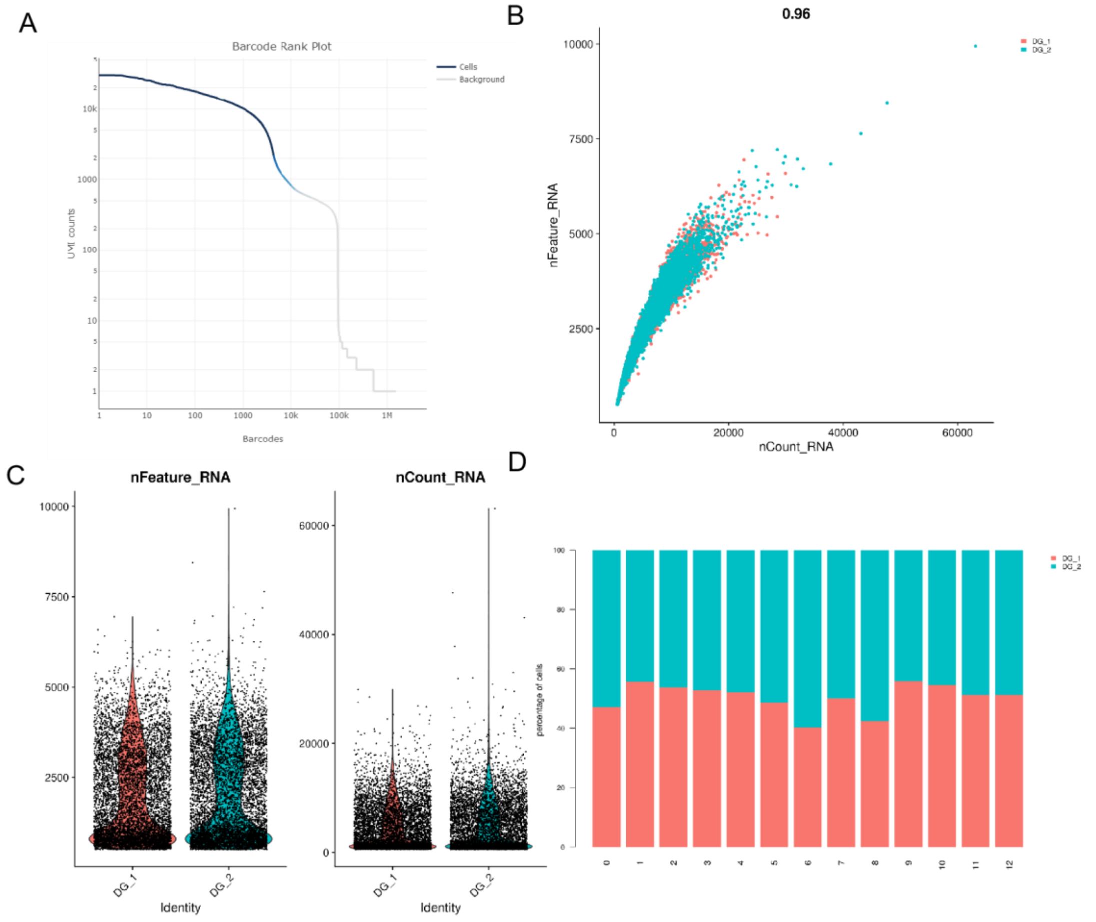

Supplemental Fig. S3 Quality control and filtering of scRNA-seq Data.

(A) Effective cell identification diagram: The barcode corresponding to the blue line is the valid cell，The barcode corresponding to the blue line is the valid cell, and the gray line is the background noise. (B) The pearson correlation coefficient between the number of UMI and the number of genes in the filtered cells of the two samples is 0.96. (C) Distribution of basic information of two sample cells after filtering: Number distribution of genes detected in a single cell of two samples (left); Number distribution of UMI detected in single cell of two samples (right). (D) Percentage of two sample cells in each cluster.

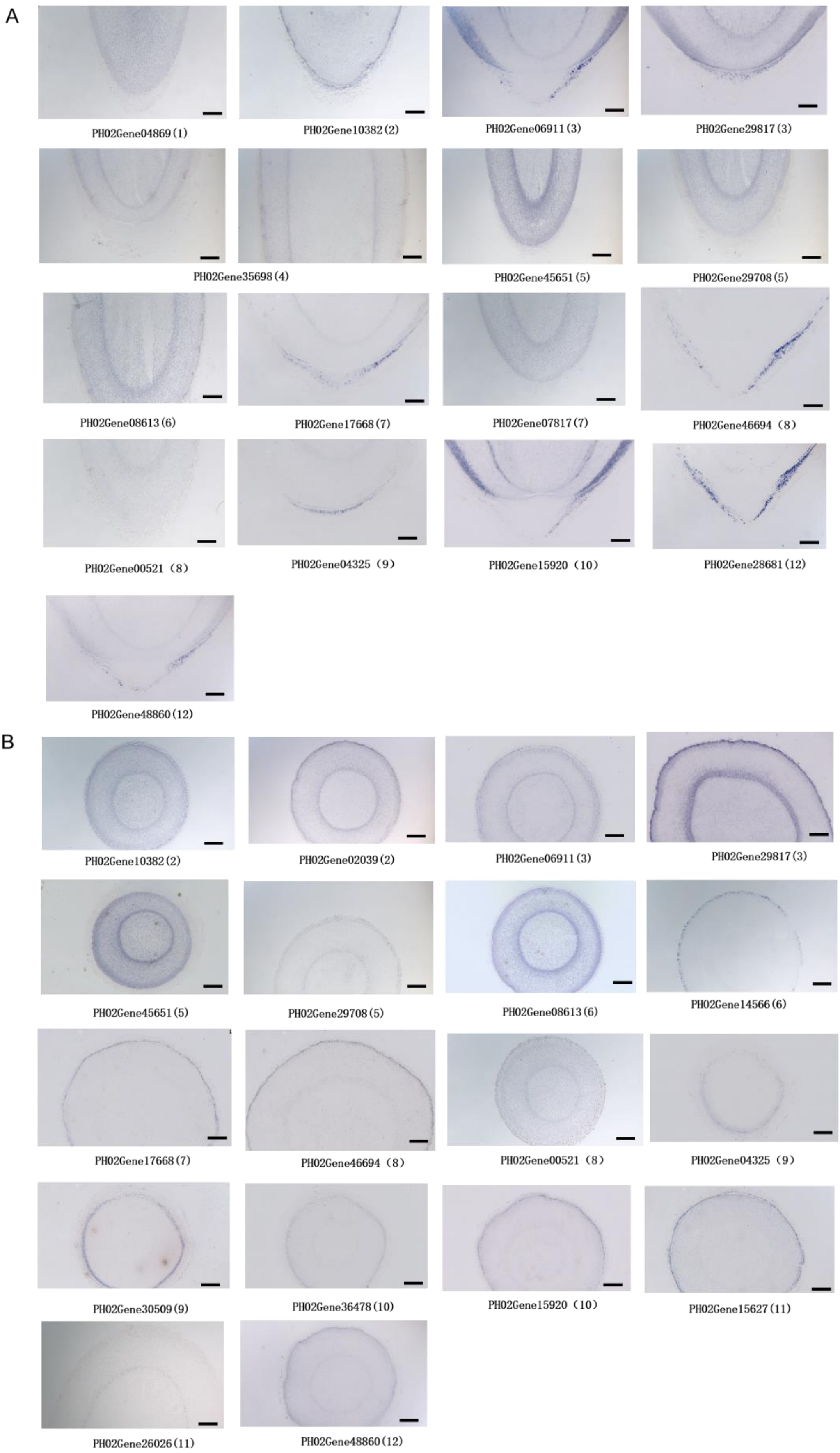

A
PH02Gene04869 (1)
PH02Gene10382 (2)
PH02Gene06911 (3)
PH02Gene29817 (3)
PH02Gene35698 (4)
PH02Gene45651 (5)
PH02Gene29708 (5)
PH02Gene08613 (6)
PH02Gene17668 (7)
PH02Gene07817 (7)
PH02Gene46694 （8）
PH02Gene00521 （8）
PH02Gene04325 （9）
PH02Gene15920 （10）
PH02Gene28681 (12)
PH02Gene48860 (12)
B
PH02Gene10382 (2)
PH02Gene02039 (2)
PH02Gene06911 (3)
PH02Gene29817 (3)
PH02Gene45651 (5)
PH02Gene29708 (5)
PH02Gene08613 (6)
PH02Gene14566 (6)
PH02Gene17668 (7)
PH02Gene46694 （8）
PH02Gene00521 （8）
PH02Gene04325 （9）
PH02Gene30509 (9)
PH02Gene36478 (10)
PH02Gene15920 （10）
PH02Gene15627 (11)
PH02Gene26026 (11)
PH02Gene48860 (12)

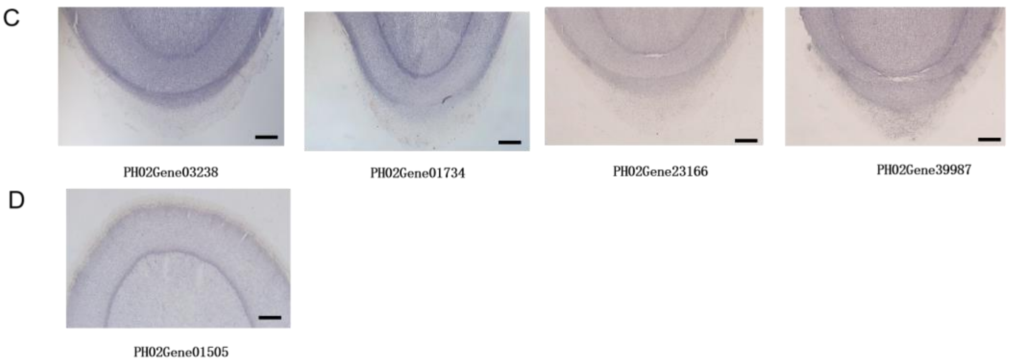

Supplemental Fig. S4 *In situ* hybridization analysis of the cell-type Marker genes and orthologs genes.

(A and B) *In situ* hybridization showing that *PH02Gene04869* belongs to 1 cell cluster, *PH02Gene10382* and *PH02Gene02039* belong to 2 cell cluster, *PH02Gene06911* and *PH02Gene29817* belong to 3 cell cluster; *PH02Gene35698* belongs to 4 cell cluster; *PH02Gene45651* and *PH02Gene29708* belong to 5 cell cluster; *PH02Gene08613* and *PH02Gene14566* belong to 6 cell cluster; *PH02Gene17668* and *PH02Gene07817* belong to 7 cell cluster; *PH02Gene46694* and *PH02Gene00521* belong to 8 cell cluster; *PH02Gene30509* and *PH02Gene04325* belong to 9 cell cluster; *PH02Gene36478* and *PH02Gene15920* belong to 10 cell cluster; *PH02Gene15627* and *PH02Gene26026* belong to 11 cell cluster; *PH02Gene28681* and *PH02Gene48860* belong to 12 cell cluster. Roots are longitudinally (A) or transversely (B) sectioned. Scale bars, 300 µm.

(C and D) 5 representatives in situ hybridization of orthologs genes showing many *Arabidopsis* marker genes were not cell-type Marker gene in Moso bamboo. Roots are longitudinally (C) or transversely (D) sectioned. Scale bars, 300 µm.

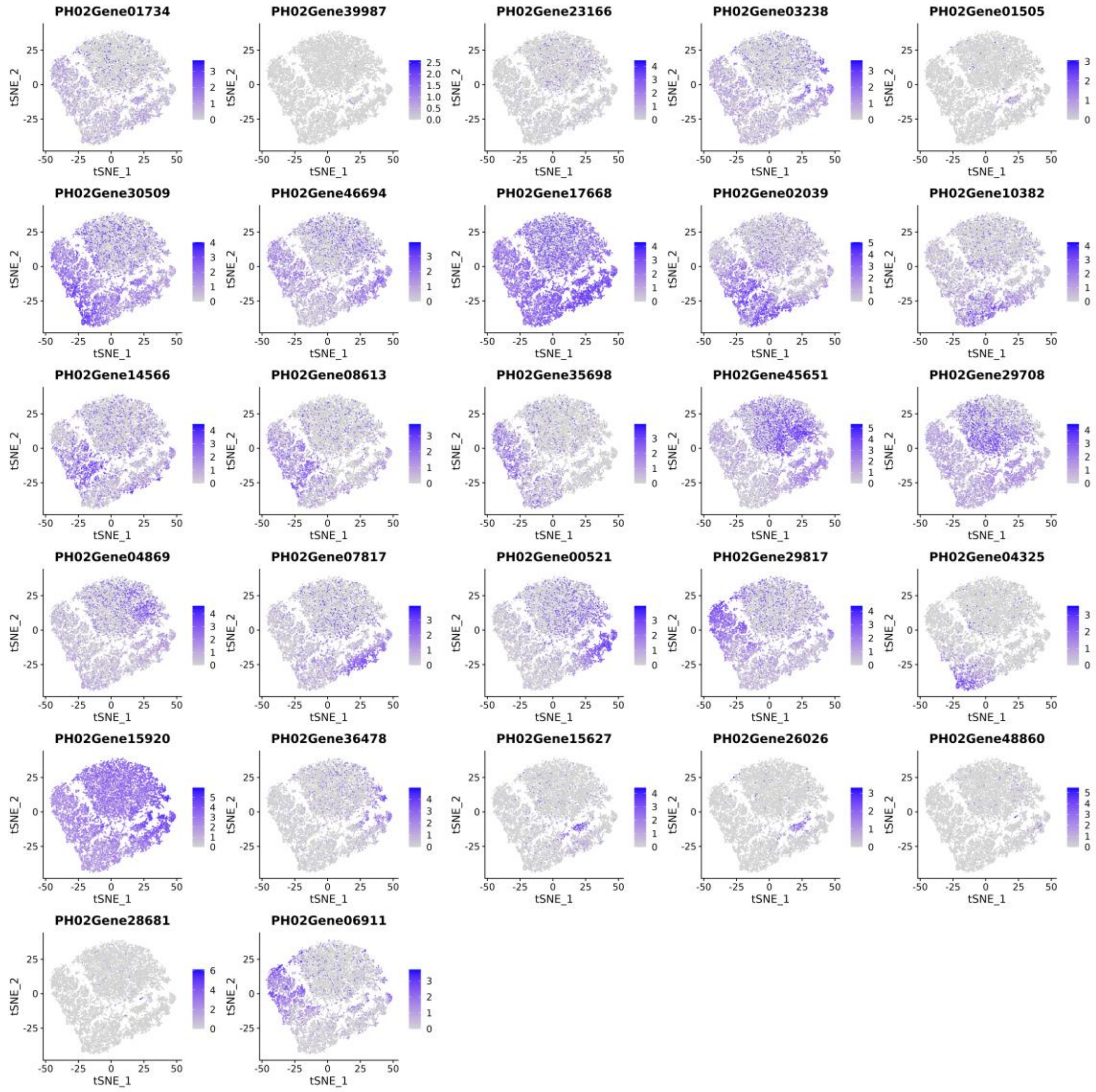

Supplemental Fig. S5 T-SNE visualization of expression of representative cell-type marker genes and orthologs genes.

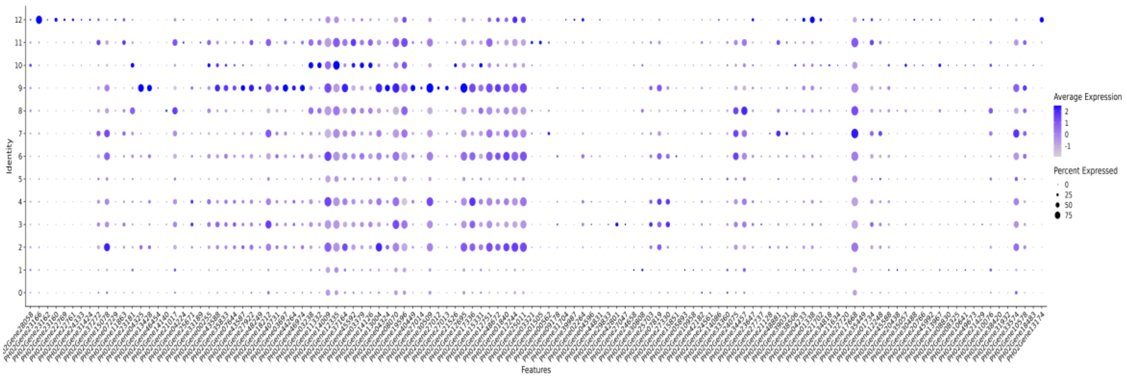

Supplemental Fig. S6 Expression of selected Arabidopsis and rice orthologs genes in each cluster. Dot diameter, proportion of cluster cells expressing a given gene; Colour bar, mean expression across cells in that cluster.

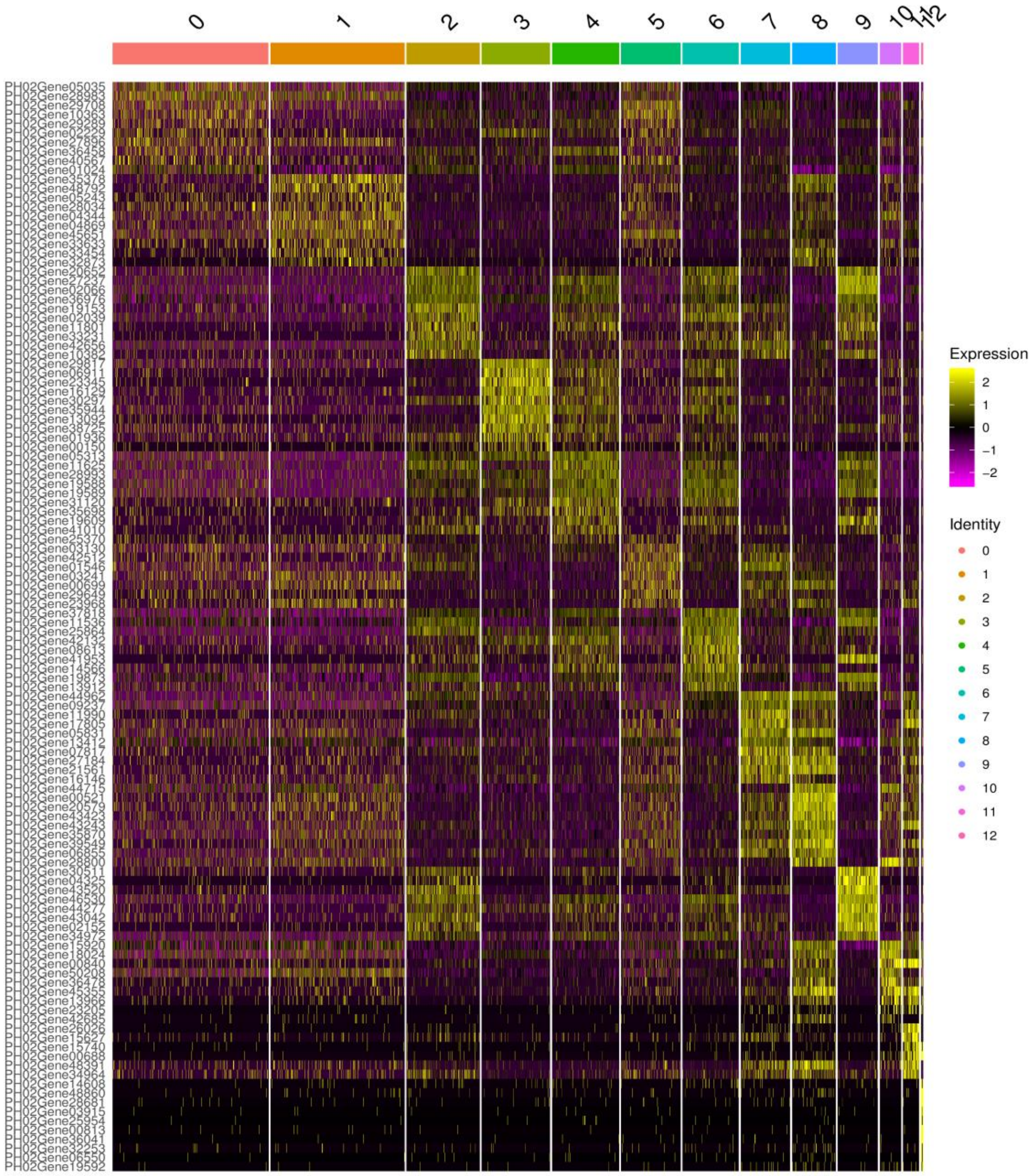

Supplemental Fig. S7 Heatmap displaying expression of top 10 cell-type Marker genes in 13 cell clusters of Moso bamboo basal root. Colour bar indicate scaled expression level.

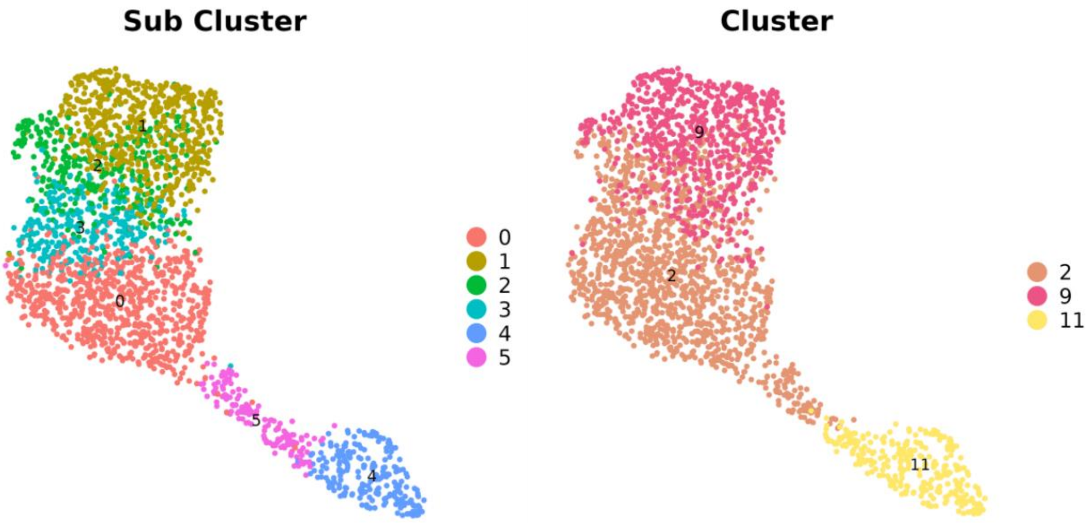

Supplemental Fig. S8 UMAP projections showing the root cap cell populations (Clusters 9, 2, 11). 0 to 5, sub-cell clusters.

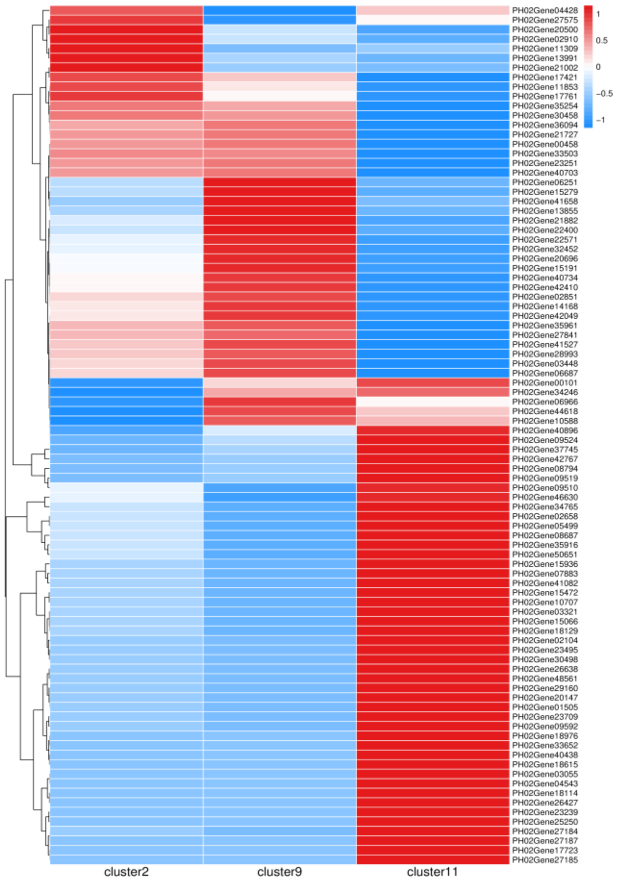

Supplemental Fig. S9 Heatmap of representative TFs expression Level of Clusters 9, 2, 11 involved in plant hormone pathway.

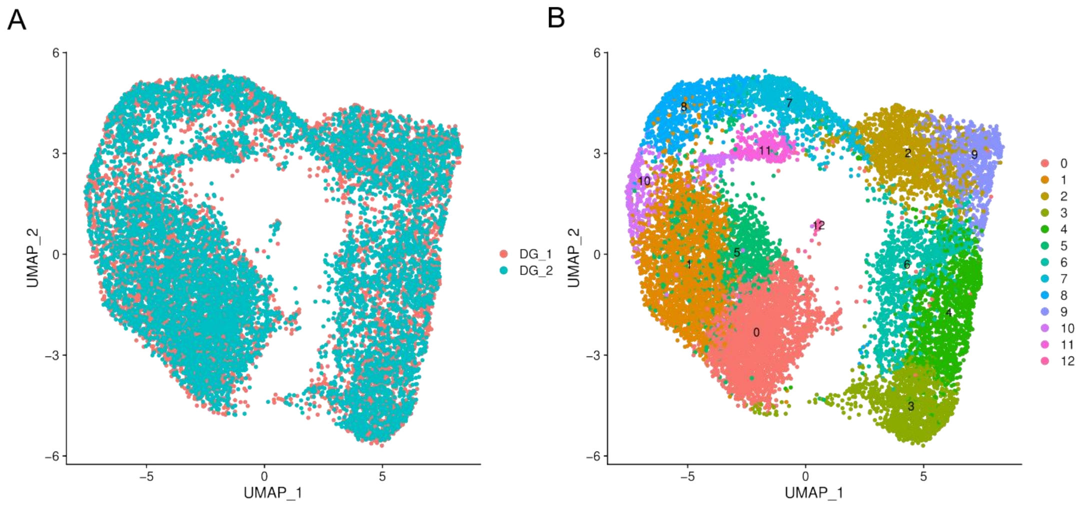

Supplemental Fig. S10 UMAP visualization of two biological replicates (A) and 13 cell clusters (B) of Moso bamboo basal root. Each dot denotes a single cell. Colors denote corresponding cell clusters as in Figure 1A.

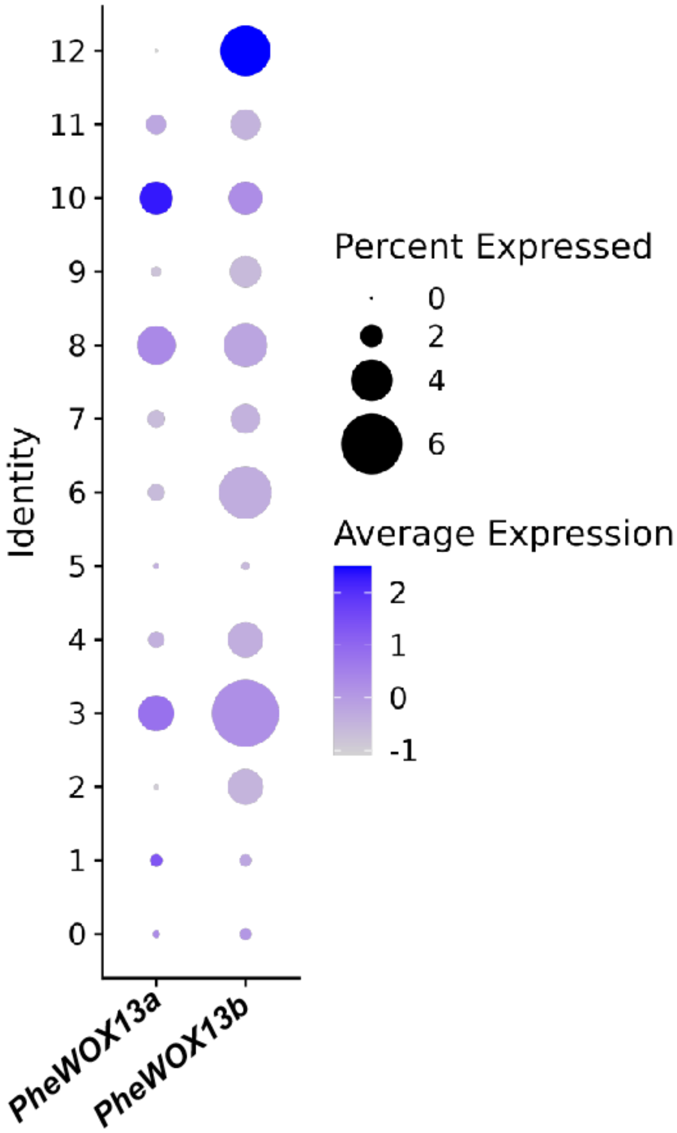

Supplemental Fig. S11 Dot plots representing scRNA-seq analysis showing expression of *PheWOX13a* and *PheWOX13b* in all cell clusters.

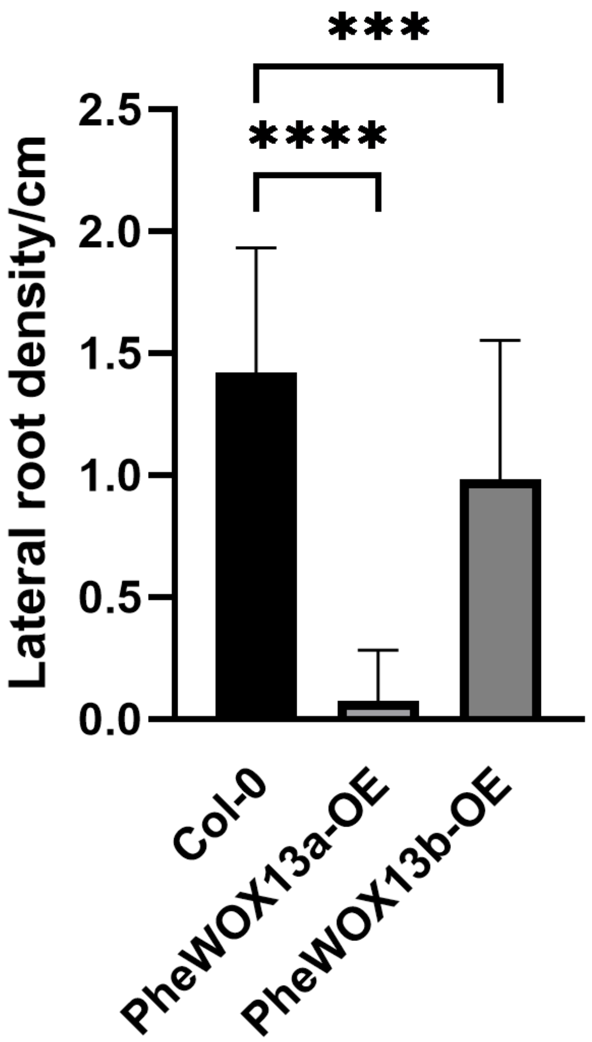

Supplemental Fig. S12 Lateral root densities in Figure 5A. Col-0, Wild type; *PheWOX13a*-OE and *PheWOX13b*-OE, Overexpression of *PheWOX13a* and *PheWOX13b* in Col-0. Data are means ± SE (n =30 roots).

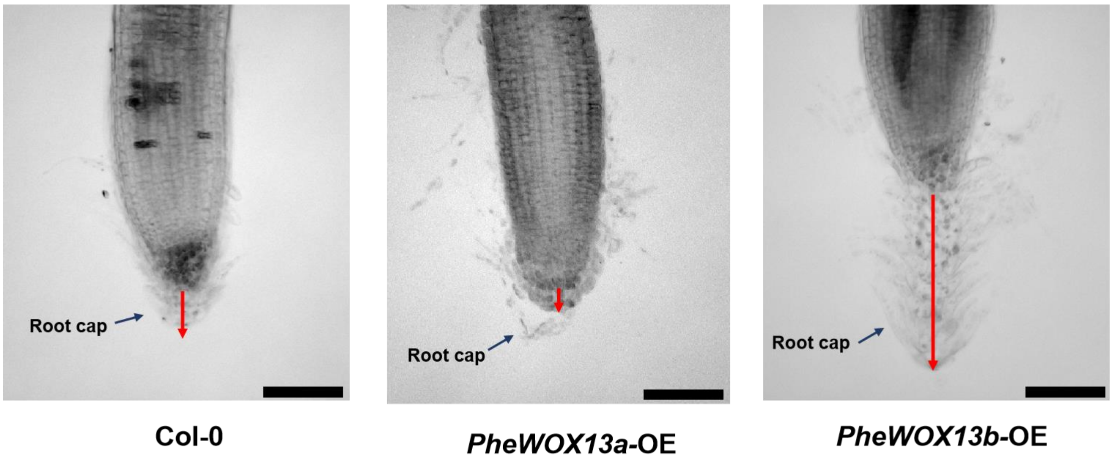

Supplemental Fig. S13 Structures of primary roots in Figure 5A. Col-0, Wild type; *PheWOX13a*-OE and *PheWOX13b*-OE, Overexpression of *PheWOX13a* and *PheWOX13b* in Col-0. ↓ Indicates the location of root cap. Scale bars, 100 μm.

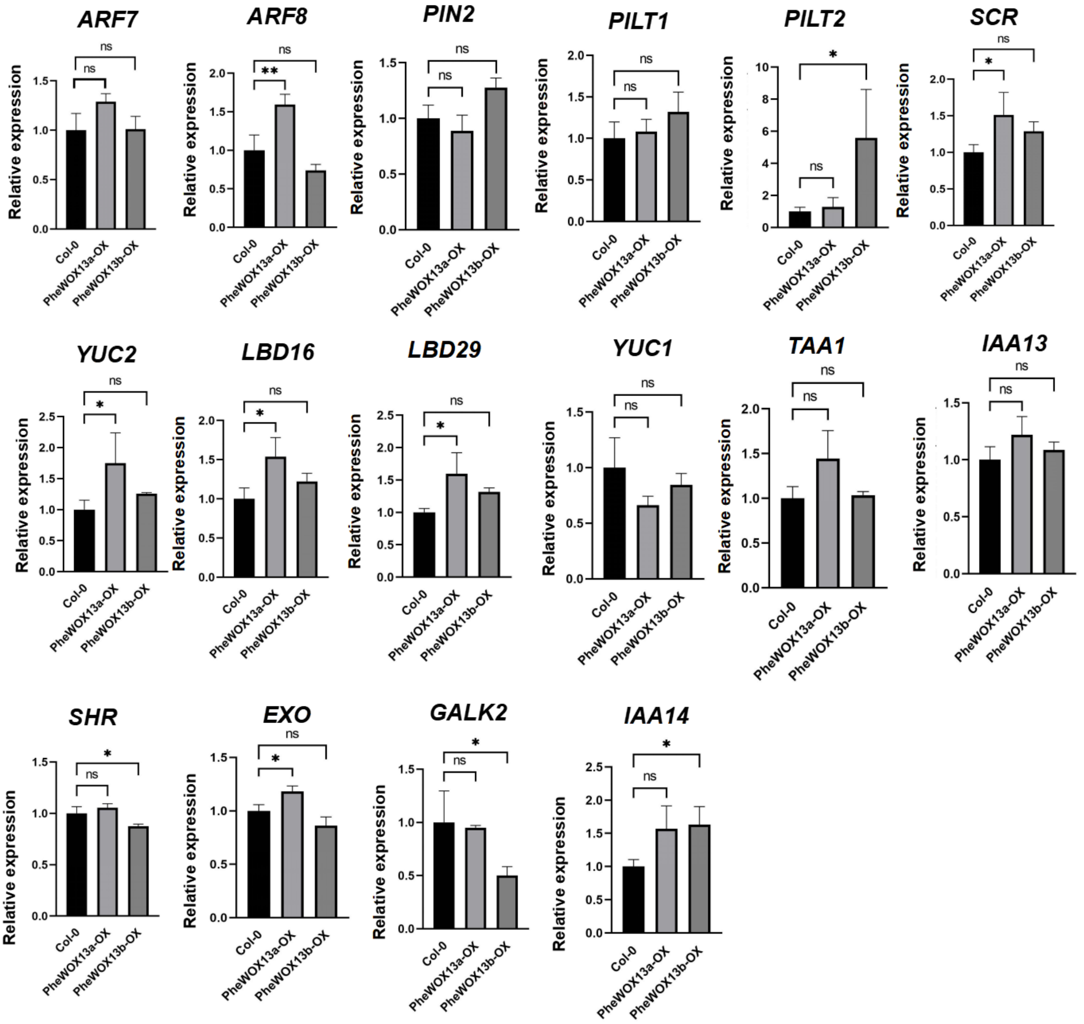

Supplemental Fig. S14 Relative expression levels of genes related to roots and lateral roots development in Col-0, *PheWOX13a*-OE and *PheWOX13b*-OE.

Col-0, Wild type; *PheWOX13a*-OE and *PheWOX13b*-OE, Overexpression of *PheWOX13a* and *PheWOX13b* in Col-0. Actin 2 served as the reference gene. Data are means ± SE (n = 3). Asterisks indicate a statistically significant difference between Col-0 and transgenic plants (T-test, *P < 0.05, **P < 0.01, ****P < 0.0001, ns, No significant difference)

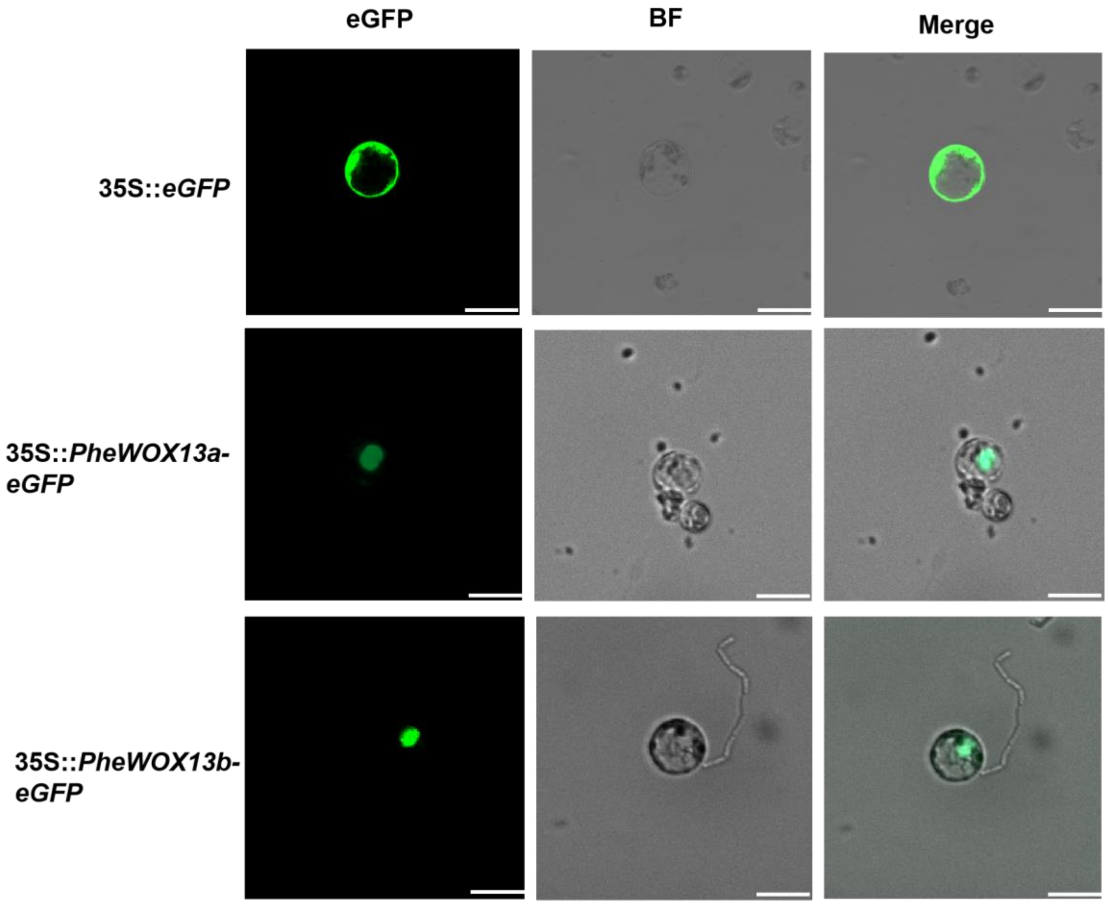

Supplemental Fig. S15 Subcellular localization of PheWOX13a-eGFP and PheWOX13b-eGFP in protoplasts of Moso bamboo leaves. eGFP, eGFP fluorescence; BF, bright field; Merge, merge of eGFP and BF images. Bars = 25 μm.

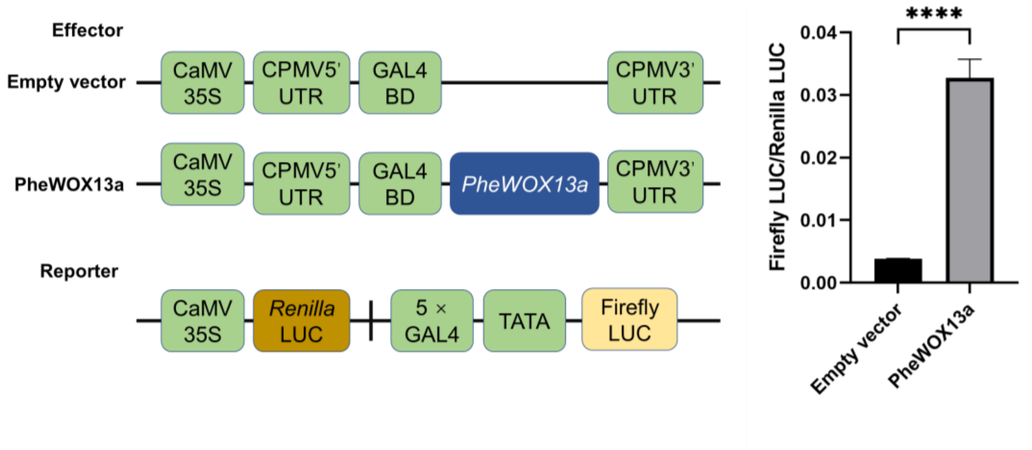

Supplemental Fig. S16 Transcription activation analysis of PheWOX13a in *Nicotiana benthamiana* leaves. Data are means ± SE (n = 3). Student's t test was used to identify significant differences compared to the empty vector control (****P < 0.0001).
